# Epithelial–Mesenchymal Transition (EMT) Induced by TGF-β in Hepatocellular Carcinoma Cells Reprograms Lipid Metabolism

**DOI:** 10.3390/ijms22115543

**Published:** 2021-05-24

**Authors:** Jitka Soukupova, Andrea Malfettone, Esther Bertran, María Isabel Hernández-Alvarez, Irene Peñuelas-Haro, Francesco Dituri, Gianluigi Giannelli, Antonio Zorzano, Isabel Fabregat

**Affiliations:** 1 TGF-β and Cancer Group, Molecular Mechanisms and Experimental Therapy in Oncology (Oncobell) Program, Bellvitge Biomedical Research Institute (IDIBELL), L’Hospitalet de Llobregat, 08908 Barcelona, Spain; jisoukupova@gmail.com (J.S.); a.malfettone@gmail.com (A.M.); ebertran@idibell.cat (E.B.); ipenuelas@idibell.cat (I.P.-H.); 2 CIBER Hepatic and Digestive Diseases (CIBERehd), Instituto de Salud Carlos III, 28029 Madrid, Spain; 3 CIBER Diabetes and Metabolic Associated Diseases (CIBERdem), Instituto de Salud Carlos III, 28029 Madrid, Spain; maria.hernandez@irbbarcelona.org (M.I.H.-A.); antonio.zorzano@irbbarcelona.org (A.Z.); 4 Biochemistry and Molecular Biomedicine Department, Universitat de Barcelona–UB, 08028 Barcelona, Spain; 5 National Institute of Gastroenterology, IRCCS “S. De Bellis” Research Hospital, Castellana Grotte, 70013 Bari, Italy; francesco.dituri@irccsdebellis.it (F.D.); gianluigi.giannelli@irccsdebellis.it (G.G.); 6 Institute for Research in Biomedicina (IRB Barcelona), 08028 Barcelona, Spain; 7 Barcelona Institute of Science and Technology (BIST), 08036 Barcelona, Spain; 8 Department of Physiological Sciences, School of Medicine and Health Sciences, Universitat de Barcelona, L’Hospitalet de Llobregat, 08907 Barcelona, Spain

**Keywords:** liver, HCC, EMT, TGF-beta, lipid metabolism, oxidative metabolism, beta oxidation

## Abstract

(1) Background: The transforming growth factor (TGF)-β plays a dual role in liver carcinogenesis. At early stages, it inhibits cell growth and induces apoptosis. However, TGF-β expression is high in advanced stages of hepatocellular carcinoma (HCC) and cells become resistant to TGF-β induced suppressor effects, responding to this cytokine undergoing epithelial–mesenchymal transition (EMT), which contributes to cell migration and invasion. Metabolic reprogramming has been established as a key hallmark of cancer. However, to consider metabolism as a therapeutic target in HCC, it is necessary to obtain a better understanding of how reprogramming occurs, which are the factors that regulate it, and how to identify the situation in a patient. Accordingly, in this work we aimed to analyze whether a process of full EMT induced by TGF-β in HCC cells induces metabolic reprogramming. (2) Methods: In vitro analysis in HCC cell lines, metabolomics and transcriptomics. (3) Results: Our findings indicate a differential metabolic switch in response to TGF-β when the HCC cells undergo a full EMT, which would favor lipolysis, increased transport and utilization of free fatty acids (FFA), decreased aerobic glycolysis and an increase in mitochondrial oxidative metabolism. (4) Conclusions: EMT induced by TGF-β in HCC cells reprograms lipid metabolism to facilitate the utilization of FFA and the entry of acetyl-CoA into the TCA cycle, to sustain the elevated requirements of energy linked to this process.

## 1. Introduction

HCC incidence has increased in the last decades and has become the second-leading cause of cancer-related death. Although novel therapies, such as new targeted agents and immunotherapies, are being rapidly incorporated [1], it is necessary to advance in the molecular mechanisms involved in this heterogeneous tumor to propose new therapeutic targets. Metabolic reprogramming has been established as a key hallmark of cancer [2]. Apart from modulation of glucose metabolism, alterations in lipid- and cholesterol-associated pathways are well recognized as contributors to liver tumor progression [3,4]. Alterations in fatty acid synthesis, β-oxidation and remodeling of lipid composition contribute to the initiation and progression of HCC [3]. A consistent body of data indicates that expression of lipogenic proteins, including FASN, is increased in human hepatocarcinogenesis and is predictive of poor prognosis of HCC patients [5]. However, HCC is very heterogeneous in etiology and genetic/epigenetic alterations, and both lipid synthesis and fatty acid oxidation (FAO) have been proposed to contribute at different moments or under different backgrounds to the progression of HCC [3]. Not only de novo synthesis, but also exogenous fatty acids play a major role in hepatocarcinogenesis [6]. Addiction to fatty acids and an increase in FAO are characteristic of β-catenin-activated HCC [7]. Indeed, to consider lipid metabolism as a therapeutic target in HCC, it is necessary to develop a better understanding about how lipid reprogramming occurs and how to identify the situation in a patient.

The TGF-β pathway plays a dual role in liver carcinogenesis [8]. At early stages, it inhibits cell growth and induces apoptosis. However, once cells become resistant to TGF-β-induced suppressor effects, they respond to this cytokine by inducing cell migration and invasion [8]. A mesenchymal, migratory and invasive phenotype in HCC cells correlates with high autocrine TGF-β expression [9]. EMT plays a role in this process, but not all the cells develop a full EMT. TGF-β may induce a partial EMT in some epithelial HCC cells, increasing the expression of mesenchymal genes but maintaining epithelial gene expression [10]. In the epithelial HCC cell line PLC/PRF/5 that responds to TGF-β by inducing migration but maintaining epithelial characteristics without induction of the transcriptomic EMT program [9], we recently reported that TGF-β reduces mitochondrial respiration, favoring glutamine anaplerosis, which contributes to convert the TCA cycle into a source of metabolites for biosynthetic purposes, including de novo FFA synthesis [11]. However, whether a similar situation may take place when TGF-β induces full EMT in HCC cells has not been explored.

Accordingly, in this work we aimed to analyze, through metabolomics and transcriptomics, whether a process of full EMT induced by TGF-β in HCC cells induces metabolic reprogramming. The results indicate that lipid catabolism is significantly affected, with increases in FAO-related processes and higher mitochondrial oxidative metabolism.

## 2. Results

### 2.1. Full EMT Induced by TGF-β in Hep3B Cells Reprograms Lipid Metabolism

In order to establish a TGF-β-induced full EMT, we selected an HCC cell line that shows a mixed epithelial–mesenchymal phenotype and that, as we previously demonstrated, induces a full EMT in response to TGF-β [10]. Indeed, acute treatment of cells with TGF-β induced an early loss of E-cadherin at the mRNA and protein level, concomitant with an increase in the expression of mesenchymal genes, such as vimentin, and up-regulation of some members of the Snail family of transcription factors, in particular, *SNAI1*, *SNAI2*, *TWIST1* and *ZEB2* (*ZEB1* did not show changes). All these changes were impaired in cells where the TGF-β Receptor I (TβRI) was stably silenced through shRNA technology (Supplementary Figure S1). After chronic treatment of Hep3B cells with TGF-β for at least two weeks (from now, TβT-Hep3B), the EMT phenotype was fully established, with loss in the expression, at the mRNA and protein levels, of E-cadherin or cytokeratin 18, characteristic proteins of an epithelial phenotype, and gain in the expression of mesenchymal proteins, such as vimentin (Supplementary Figure S2A,B). Furthermore, cells presented increased expression of the EMT-related transcription factors *SNAI1*, *SNAI2* or *ZEB2* (Supplementary Figure S2B), and augmented cell migration (Supplementary Figure S2C). Here we have used this cell model to perform combined metabolomic profiling and gene expression analyses in order to understand the metabolic changes that may occur during the TGF-β-induced EMT in HCC.

**Figure 1:**
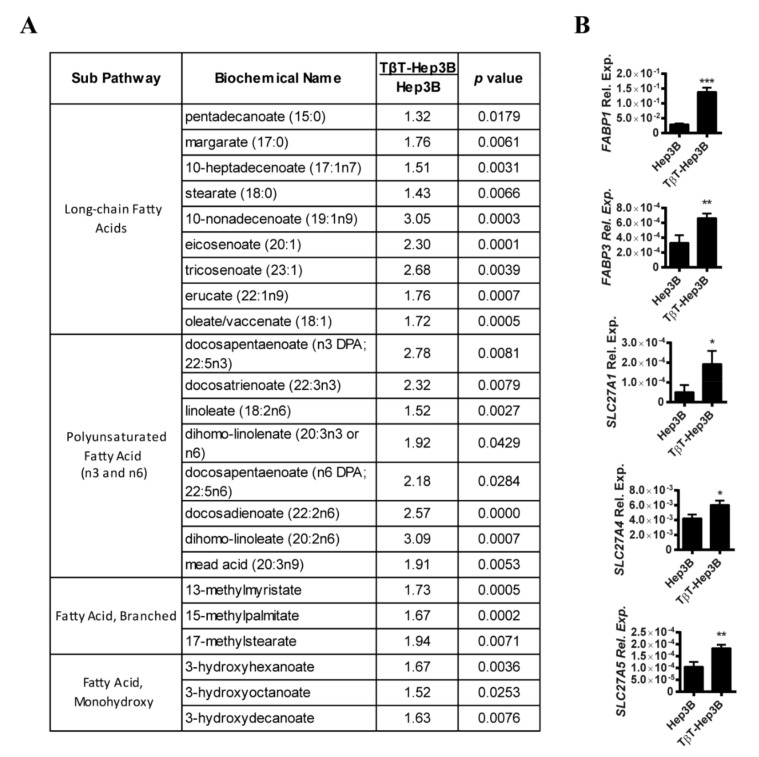
Chronic TGF-β treatment in Hep3B cells led to an increase in fatty acid content. (**A**) Fatty acid metabolites of TβT-Hep3B compared to Hep3B presented in fold. Welch’s two-sample *t*-test was used to identify biochemicals that differed significantly between experimental groups (*n* = 5 for each group, *p* value indicated in the right column). (**B**) Expression of genes related to fatty acid transport that changed significantly between Hep3B and TβT-Hep3B. Mean ± SD (*n* = 3). * *p* < 0.05, ** *p* < 0.01, *** *p* < 0.001.

Metabolomic analysis of Hep3B cells versus TβT-Hep3B revealed 151 significantly altered metabolites. Interestingly, by pathway set enrichment analysis we found out that the most important changes occurred in lipid metabolism. Unsupervised hierarchical clustering was performed using Euclidean distance and showed significant differences between Hep3B and TβT-Hep3B (Supplementary Figure S3). Indeed, levels of a relevant number of FFA were increased in TβT-Hep3B, including long-chain, polyunsaturated, branched and monohydroxy fatty acids (Figure 1A, the full list of utilized fatty acid metabolites in Supplementary Table S1). The accumulation of FFA seems not to be related to increased fatty acid synthesis, as the expression of genes related to fatty acid synthesis, such as fatty acid synthase (*FASN*), acetyl-CoA carboxylase alpha (*ACACA*) and acetyl-CoA carboxylase beta (*ACACB*), was not changed (full list of utilized genes in Supplementary Table S2). Accumulation of FFA could be also related to an increase in their transport. In this sense, the transporters of fatty acids, including various members of the family of fatty acid-binding proteins (FABPs) and the family of long-chain fatty acid transporters, solute carrier family 27, were upregulated (Figure 1B).

**Figure 2:**
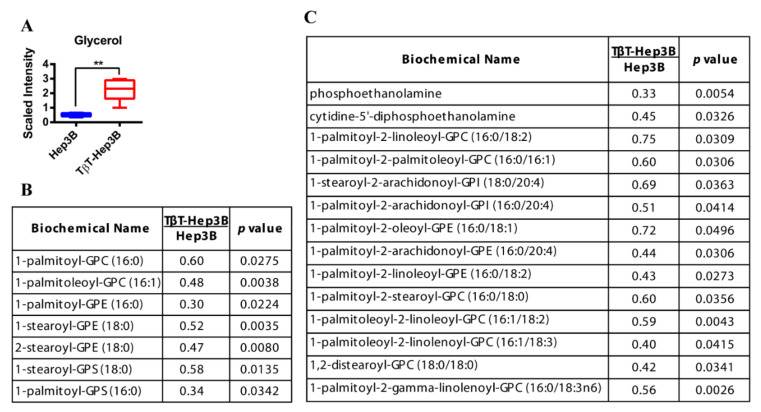
Increased glycerol levels and decreased lysolipids and phospholipids in TβT-Hep3B cells. (**A**) Level of glycerol is depicted by a box plot with whiskers (min to max). Welch’s two-sample *t*-test was used to identify biochemicals that differed significantly between experimental groups (*n* = 5 for each group). ** *p* < 0.01. (**B**,**C**) Levels of lysolipids (**B**) and phospholipids (**C**) of TβT-Hep3 compared to Hep3B presented in fold. Welch’s two-sample *t*-test was used to identify biochemicals that differed significantly between experimental groups (*n* = 5 for each group, *p*-value indicated in the right column).

The levels of glycerol were significantly increased (4.35-fold) in TβT-Hep3B cells (Figure 2A), which would suggest elevated FFA release from stores. Confirming this hypothesis, levels of monoacylglycerols and diacylglycerols were in general decreased in TβT-Hep3B, being clearly significant in some cases, such as palmitoleoyl-oleoyl-glycerol or palmitoyl-linoleoyl-glycerol (Supplementary Table S1), and the expression of *LIPE* (Lipase E or hormone-sensitive lipase) was increased in TβT-Hep3B cells (2.94 fold increase, *p* = 0.07). Furthermore, the levels of various lysolipids were decreased in TβT-Hep3B cells, including 1-acyl glycerophosphocholines, 1-alkenyl glycerophosphocholines, 1(2)-acyl glycerophosphoethanolamines or 1-acyl glycerophosphatidyserines (Figure 2B). Lysolipids were also decreased in TβT-Hep3B, including those bound to glycerophosphocholines, glycerophosphoethanolamines, glycerophosphatidyserines and glycerophosphoinositols (Figure 2C). Overall, these findings indicate that increased lipid turnover could be also responsible for the larger pool of FFA and glycerol.

### 2.2. EMT Induced by TGF-β in HCC Cells Correlates with Increased FFA β-Oxidation

**Figure 3:**
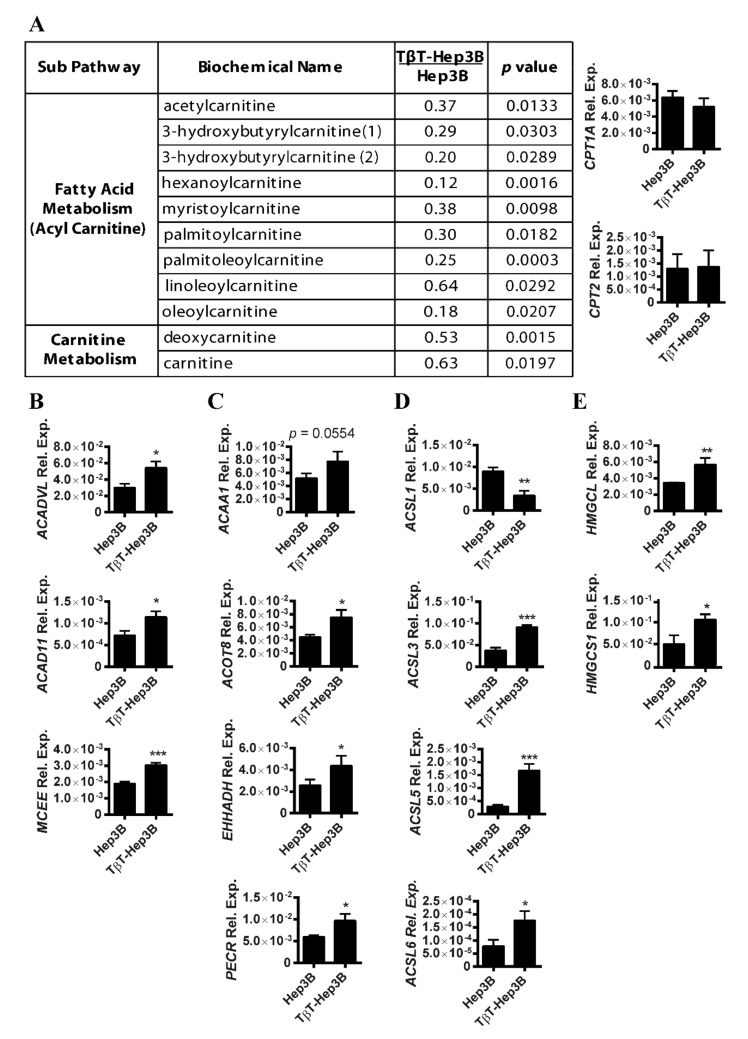
Decrease in carnitines and acyl carnitines in TβT-Hep3B correlates with an increased expression of β-oxidation genes. (**A**) Left: metabolites of carnitine pathway of TβT-Hep3B compared to Hep3B presented in fold. Welch’s two-sample *t*-test was used to identify biochemicals that differed significantly between experimental groups (*n* = 5 for each group, *p* value indicated in the right column). Right: expression of *CPT1A* and *CPT2*. Mean ± SD (*n* = 3). (**B**–**E**) Expression of genes related to mitochondrial β-oxidation (**B**), peroxisomal β-oxidation (**C**), ACSL family members (**D**) and ketogenesis (**E**). Mean ± SD (*n* = 3). * *p* < 0.05, ** *p* < 0.01, *** *p* < 0.001.

Changes in lipid turnover could be related to the release of FFA to be used as a source of energy. Carnitines are responsible for FFA mobilization and transport for β-oxidation to the mitochondria. We observed that carnitine levels and carnitine-conjugated lipids were significantly decreased in TβT-Hep3B cells, including acetylcarnitine, 3-hydroxybutyrylcarnitine, hexanoylcarnitine, myristoylcarnitine, palmitoycarnitine and others. This decrease in carnitine derivatives could be a sign of increased utilization or decreased transport. However, the expression of genes related to fatty acid transport via carnitines, such as *CPT1A* and *CPT2*, was not affected (Figure 3A). The decrease in carnitines and carnitine-conjugated lipids might, therefore, be a result of an increased utilization of carnitine derivatives for β-oxidation.

Confirming this hypothesis, the expression of genes coding enzymes responsible for the dehydrogenation of long- and very long-chain fatty acids, such as acyl-CoA dehydrogenase long chain (*ACADL*), acyl-CoA dehydrogenase very long chain (*ACADVL*) and acyl-CoA dehydrogenase family member 11 (*ACAD11*), was increased in TβT-Hep3B cells, as well as mitochondrial methylmalonyl-CoA epimerase (*MCEE*), an enzyme responsible for the final degradation of odd-chain fatty acids (Figure 3B). The β-oxidation of FFA with a long chain (C > 22) occurs preferentially in peroxisomes (peroxisomal β-oxidation). Indeed, the expression of many enzymes related to peroxisomal β-oxidation was also upregulated in TβT-Hep3B cells, such as acetyl-CoA acyltransferase 1 (*ACAA1*), acyl-CoA thioesterase 8 (*ACOT8*), enoyl-CoA hydratase (*EHHADH*) and a peroxisomal trans-2 enoyl CoA reductase (*PECR*) (Figure 3C). Interestingly, a family of acylCoA synthetase proteins (*ACSLs*) responsible for converting long-chain fatty acids to acylCoA for mitochondrial and peroxisomal β-oxidation was dysregulated in TβT-Hep3B cells (Figure 3D). In conclusion, TGF-β treatment in Hep3B led to an increase in β-oxidation of long-chain fatty acids, both in mitochondria and peroxisomes. The overexpression of genes related to ketogenesis was also observed, such as 3-hydroxymethyl-3-methylglutaryl-CoA lyase (*HMGCL*) (Figure 3E), as well as the cytosolic hydroxymethyl-3-methylglutaryl-CoA synthase (*HMGCS1*), involved in the cytosolic synthesis of HMGCoA.

### 2.3. Metabolomic Changes after Silencing TβRI in a Mesenchymal HCC Cell Line

To reinforce these results, we decided to silence the TGF-β Receptor I (TβRI) in an HCC cell line, SNU449, that shows a full mesenchymal phenotype, due to the high autocrine expression of TGF-β [9]. When the TβRI was stably silenced in these cells, by using specific shRNA, the expression of EMT-related transcription factors, such as *SNAI1*, *TWIST1* or *ZEB1*, was significantly down-regulated (Supplementary Figure S4A). Although the decrease in the level of vimentin (as a mesenchymal marker) was modest, its organization clearly reflected phenotypic changes, with a less spindle-like, more epithelial-like aspect. This observation was confirmed through staining of F-Actin with phalloidin, which reflected the loss of stress fibers, or the appearance of tight junctions by immunostaining with ZO-1 (Supplementary Figure S4B). Interestingly, transcriptomic and phenotypic changes observed by TβRI silencing correlated with a decrease in cell migratory capacity (Supplementary Figure S4C).

**Figure 4:**
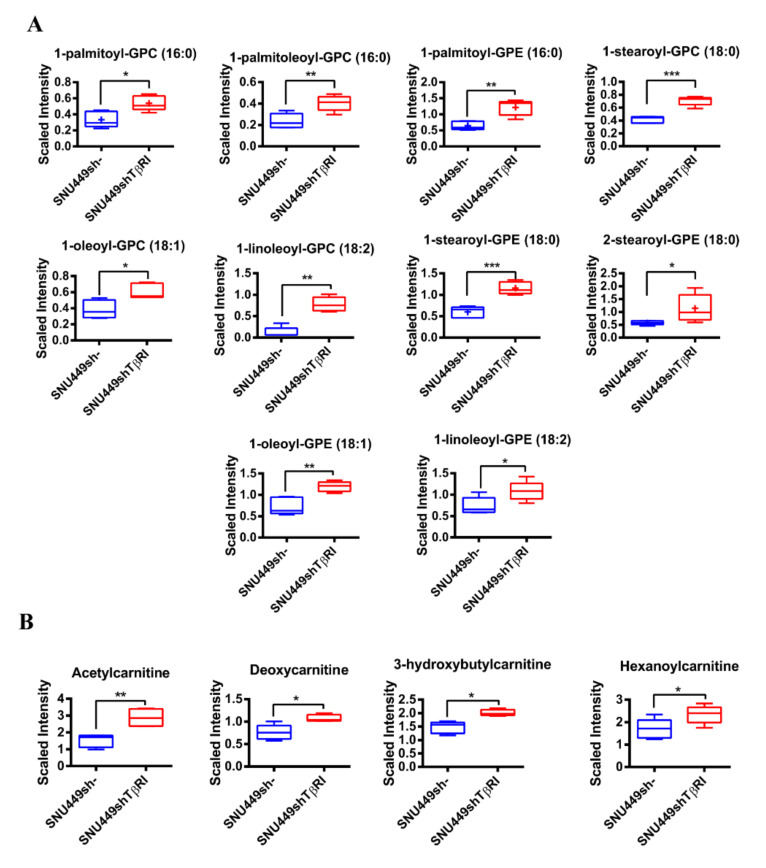
Lipidomic analysis in SNU449shTβRI cells. Levels of lysolipids (**A**) and carnitine-related metabolites (**B**) are depicted by a box plot with whiskers (min to max). Welch’s two-sample *t*-test was used to identify biochemicals that differed significantly between experimental groups (*n* = 5 for each group). * *p* < 0.05, ** *p* < 0.01, *** *p* < 0.001.

Metabolomic analysis of these cells revealed opposite changes to those observed in TGF-β-treated Hep3B cells. Indeed, levels of many lysolipids were increased, including 1-acyl glycerophosphocholines, 1-alkenyl glycerophosphocholines and 1-acyl glycerophosphoethanolamines (Figure 4A). Levels of carnitines and acylcarnitines were increased, including acetylcarnitine, deoxycarnitine, 3-hydroxybutylcarnitine and hexanoylcarnitine, which would reflect accumulation due to a decrease in FFA β-oxidation (Figure 4B).

### 2.4. TβT-Hep3B Shows Increased Mitochondrial Oxidative Metabolism

**Figure 5:**
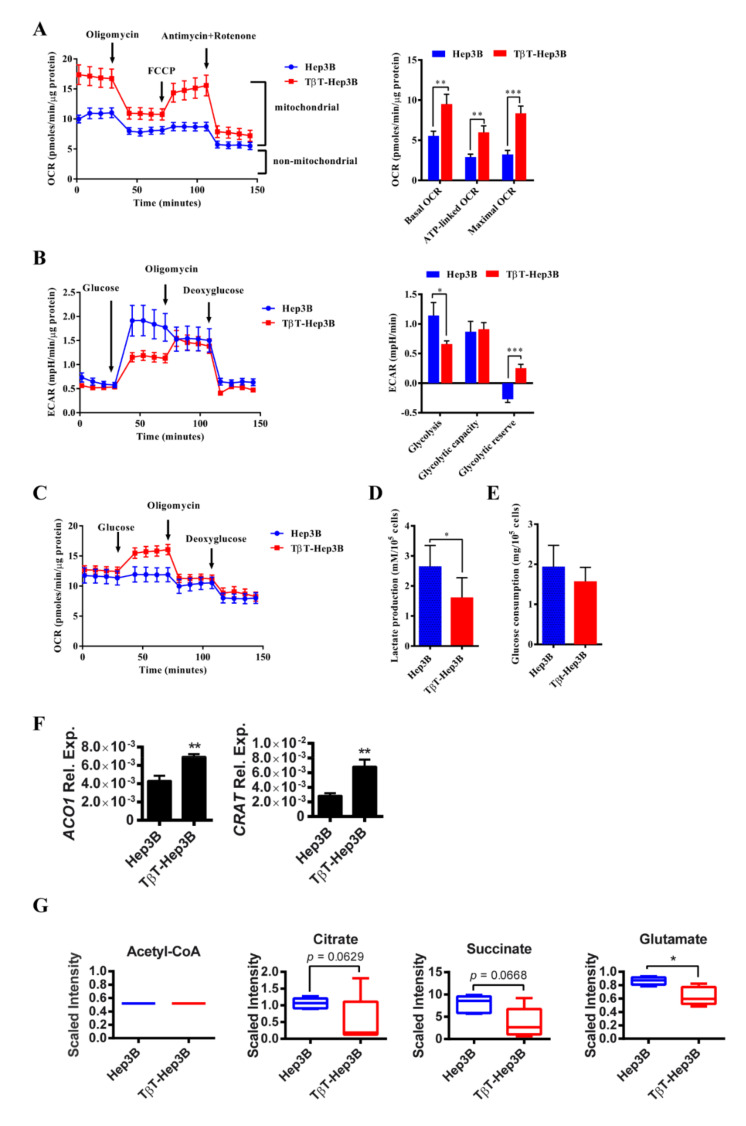
A switch from glycolysis to OXPHOS is observed in TβT-Hep3B cells. (**A**) OCR in Hep3B and TβT-Hep3B incubated 30 min prior to experiment in XF assay medium supplemented with 5 mM glucose and 2 mM glutamine and consecutively injected with oligomycin (1 μM), FCCP (0.5 μM), antimycin (1 μM) and rotenone (1 μM). Continuous OCR values (pmoles/min/μg protein) are shown. Mitochondrial functions were analyzed as explained in Supplementary Materials and Methods. Mean ± SEM (*n* = 9 from three independent experiments). ** *p* < 0.01, *** *p* < 0.001. (**B**,**C**) Hep3B and TβT-Hep3B incubated 30 min prior to experiment in XF assay medium supplemented with 2 mM glutamine and consecutively injected with glucose (10 mM), oligomycin (1 μM) and deoxyglucose (50 mM). Continuous ECAR (**B**) and OCR (**c**) values are shown. Glycolytic functions were utilized, as explained in Supplementary Materials and Methods. Mean ± SEM (*n* = 9 from three independent experiments). * *p* < 0.05, *** *p* < 0.001. (**D**) Lactate production was measured after 48 h of cell culture in DMEM supplemented with 10% FBS and normalized to cell number. Mean ± SD (*n* = 3). * *p* < 0.05. (**E**) Glucose consumption was measured after 48 h of cell culture in DMEM supplemented with 10% FBS and normalized to cell number. Mean ± SD (*n* = 3). (**F**) The expression of *ACO1* and *CRAT*. Mean ± SD (*n* = 3). ** *p* < 0.01. (**G**) Metabolites from TCA pathway are depicted by a box plot with whiskers (min to max). Welch’s two-sample *t*-test was used to identify biochemicals that differed significantly between experimental groups (*n* = 5 for each group). * *p* < 0.05.

To explore the consequences of this lipid metabolic reprogramming on oxidative mitochondrial metabolism, we analyzed the oxygen consumption rates (OCR) during sequential treatment with compounds that modulate mitochondrial activity using a Seahorse apparatus (more details in the Methods section). TβT-Hep3B showed an increased basal OCR, as well as ATP-linked OCR and maximal OCR induced by FCCP, indicative of an increased oxidative phosphorylation (OXPHOS) in those cells (Figure 5A).

Consequently, we analyzed the aerobic glycolytic activity (ECAR: extracellular acidification rate) in cells cultivated prior to the experiment in glucose-free XF Seahorse medium submitted to a sequential treatment with compounds that modulate glycolytic activity. An apparent decrease in glycolysis was observed in TβT-Hep3B. Moreover, Hep3B cells did not possess a glycolytic reserve capacity, because oligomycin (ATP synthase inhibitor that permits the readout of maximal ECAR) was not able to increase ECAR. However, TβT-Hep3B cells were able to increase glycolysis when OXPHOS was inhibited (Figure 5B). Interestingly, simultaneous analysis of OCR revealed that Hep3B cells deprived of glucose increased ECAR when glucose was injected; however, TβT-Hep3B cells increased OCR (Figure 5C). Lactate production after 48 h in cell culture was also decreased in TβT-Hep3B cells (Figure 5D). Glucose uptake between Hep3B and TβT-Hep3B cells was similar, suggesting that only the glucose destination was different between those groups (Figure 5E).

Among TCA cycle enzymes, only aconitase 1 (*ACO1*) was upregulated. Interestingly, carnitine-O-acetyltransferase (*CRAT*), an enzyme responsible for the transport of acetyl-CoA to mitochondria, was upregulated in TβT-Hep3B cells (Figure 5F). Even though acetyl-CoA levels were unchanged in TβT-Hep3B cells, we detected a tendency of decreased citrate and succinate levels and a significant decrease in glutamate in TβT-Hep3B cells, suggesting an activated TCA cycle (Figure 5G).

Overall, these results suggest that TGF-β-induced EMT in HCC cells correlates with increased activation of TCA cycle and utilization of OXPHOS for energy production, as well as a decreased level of aerobic glycolysis.

## 3. Discussion

The metabolism of tumors is different from that in normal tissues, to favor maximal use of the resources and the generation of an adequate energy balance to allow cancer cells to proliferate and survive in a competitive, sometimes hostile, microenvironment. The metabolic reprogramming of tumor cells can induce metabolic dependencies that may be exploited therapeutically [12]. Although alterations in fatty acid metabolism in liver cancer cells have received less attention when compared to other metabolic alterations, such as glucose or glutamine metabolism, recent data have pointed out the importance of lipid metabolic reprogramming in HCC, which is associated in a complex manner with the status of other metabolic pathways, such as glucose metabolism [13]. However, further studies are necessary to obtain a deeper understanding of the elements that regulate this metabolic reprogramming to apply the information in the design of new clinical settings.

TGF-β plays pro-tumorigenic roles in the liver tumor cells, due to its capacity to induce migration and invasion. Although many mechanisms are involved in these effects, the induction of EMT is one of the well-orchestrated processes that mediate the loss of cell–cell contacts and cytoskeleton remodeling, with cells acquiring a mesenchymal phenotype and migratory and invasive properties [14]. In parallel, TGF-β also induces changes in tumor cell plasticity, conferring the properties of a migratory tumor-initiating cell (TIC) [15]. Recent advances have focused on the identification of clinically relevant mechanisms that impinge on important EMT transcription factors, as well as on miRNAs causing EMT signatures and HCC progression. However, a lack of knowledge exists about how TGF-β modulates metabolism when inducing EMT. Here, we have used metabolomic and transcriptomic analyses to determine how an HCC cell line that responds to TGF-β-inducing EMT re-modulates its metabolism.

Despite the roles that lipid catabolism and mitochondrial/peroxisomal FAO could play in cancer metabolism, the biological mechanisms and therapeutic intervention have not been well defined yet. The results presented here indicate that the major metabolic changes observed in HCC cells that have undergone EMT after chronic treatment with TGF-β include relevant changes in lipid turnover and an increase in lipid catabolism, which correlate with major OXPHOS capacity and a decrease in aerobic glycolysis. Peroxisomal and mitochondrial FAO appear to be increased, which would facilitate the use of carbon resources in an efficient TCA cycle. The results contrast with different findings that correlate HCC with a higher capacity of de novo FFA synthesis, through increased expression of *FASN* and an anerobic lipid metabolism [5]. However, here we have utilized a situation that may occur in a percentage of patients in which an increase in *TGFB1* expression correlates with a full EMT [10]. Different reports in the literature propose specific changes in lipid metabolism associated with EMT in tumors. Indeed, Jiang and cols. elegantly described that EMT induced by TGF-β in non-small cell lung carcinoma cells is accompanied by a coordinated reduction in the levels of enzymes involved in the conversion of glucose into FFA and concomitant enhanced respiration [16]. Under those conditions, Snail, a key regulator of the EMT transcription program, mediated the suppression of the lipogenic program. In addition, stable FASN knock-down was enough to induce EMT and to stimulate migration and extravasation in vitro [16]. In colon cancer cells, a cooperative metabolic network comprising overexpression of the Acyl-CoA synthetases ACSL1, ACSL4 and the related enzyme SCD induces EMT and increases cellular migration and invasion [17]. A recent study also identified Snail as an inducer of FFA oxidation in breast cancer cells [18].

In general terms, it has been proposed that EMT-committed cancer cells could rely on an aerobic glycolytic metabolism or could turn toward the more efficient OXPHOS, depending on tumor type, tumor stage or EMT stimulus [19]. However, notably, the expression of the lipogenic enzymes FASN and ACC are down-regulated in circulating breast cancer cells, which exhibit enhanced mitochondrial respiration that fuels cancer cell motility [20]. EMT cells undergo oxidative shock upon intravasation of blood cells, followed by attacks from the immune system and requirement of anchorage-independent survival [21]. Switching their glycolytic metabolism towards a hybrid, more oxidative state would allow them to adapt to the environment, whereupon they find a new metastatic niche [22]. In this case, OXPHOS is increased and FAO can serve as an energy source. This metabolic change toward a lipid catabolic signature may be reversible upon withdrawal of the EMT stimulus, as we also observe in a mesenchymal HCC cell line that after silencing TβRI partially moves to a more epithelial-like phenotype. In this way, metastatic cells may re-activate lipid anabolism upon arrival to secondary sites, to sustain rapid cell proliferation. Concomitant with the EMT process induced by TGF-β in the HCC cells, here we observe an increased expression of fatty acid transporters, including various members of the family of fatty acid-binding proteins (*FABPs*) and the family of long-chain fatty acid transporters, solute carrier family 27 (SLC27), suggesting an increased fatty acid uptake that correlates with the higher level of intracellular FFA. It is interesting to mention a recent study in HCC demonstrating that the uptake of FFA via CD36 is associated with EMT. In those studies, the treatment of human liver cancer cell lines with FFAs exacerbated the EMT phenotype, whereas chemical inhibition of CD36 mitigated these effects [23]. Indeed, the observation that dysregulation of metabolism, in some circumstances, drives EMT, led to the proposal that metabolism could act as a core component of the signaling cascade elicited by the EMT [24].

TGF-β is a pleiotropic cytokine that induces different molecular mechanisms and diverse effects depending on the type of cell, cell context and microenvironment [25]. For these reasons, when analyzing its effects on metabolism, stimulation of both glycolysis and mitochondrial respiration may be observed, and both an increase in lipid synthesis or the opposite lipid degradation have been also reported [26]. The differences may be related not only to the cell type, but also to the specific response of the cells. Once HCC cells overcome TGF-β-induced suppressor effects, they respond to it by inducing cell migration and invasion. However, the mechanism is not always identical, and partial EMT processes [10] or even epithelial–ameboid transitions [27] may take place. In fact, as mentioned in the introduction section, a previous study from our group proposed that TGF-β reduces mitochondrial respiration, favoring aerobic glycolysis and glutamine anaplerosis in the PLC/PRF/5 HCC cell line, which does not undergo a full EMT in response to TGF-β [11], or even may undergo epithelial–ameboid transition under determined conditions [27]. Here, our results indicate a different metabolic switch in response to TGF-β when the HCC cell undergoes a full EMT, which would favor lipolysis, FFA transport and utilization and decreased aerobic glycolysis. These effects might be ascribed to the increase in the entry of acetyl-CoA into the TCA cycle and OXPHOS instead of lipogenesis, generating sufficient ATP for this type of cancer cell migration, as previously suggested [16,28].

In conclusion, here we describe specific metabolic changes mediated by TGF-β when it undergoes EMT in HCC cells. The results highlight the relevance of lipid turnover in liver tumor cells and suggest that lipid metabolism is adapted to the specific cell requirements, which could be different for proliferation than for migration/invasion. These findings also provide new perspectives to consider FFA β-oxidation and OXPHOS in targeted therapeutic strategies to limit HCC metastasis.

## 4. Materials and Methods

### 4.1. Cell Culture

Two different HCC cell lines were used in the study: Hep3B, with epithelial characteristics and moderate autocrine expression of TGF-β, and SNU449 cells, which show a mesenchymal phenotype and higher autocrine TGF-β expression [10,11]. Both cell lines were obtained from the European Collection of Cell Cultures (ECACC) and were never used in the laboratory for longer than four months after receipt or resuscitation. Cells were maintained in DMEM media (Lonza, Basel, Switzerland) supplemented with 10% FBS (Sera Laboratories International Ltd., West Sussex, UK), penicillin (100 U/mL), streptomycin (100 μg/mL) and amphotericin (2.5 μg/mL) and L-glutamine (2 mM). They were maintained in a humidified atmosphere at 37 °C, 5% CO_2_. For chronic TGF-β treatment, human recombinant TGF–β1 (Calbiochem, La Jolla, USA) was used at 2 ng/mL and replaced every 48 h. Stable transfection of the TGF-β Receptor I (*TβRI*) was performed as previously published [11]. Cells were observed under an Olympus 70iX microscope. Further information about targeted knock-down of *TβRI* and immunofluorescence analysis is included in Supplementary Materials and Methods .

### 4.2. Metabolic Profiling

Cell samples (100 μL of cell pellet, five replicates for each group) were submitted for metabolic profiling to Metabolon Inc. In brief, samples were extracted in methanol and the resulting extract was divided into five fractions: two for analysis by two separate reverse phase (RP)/UPLC-MS/MS methods with positive ion mode electrospray ionization (ESI), one for analysis by RP/UPLC-MS/MS with negative ion mode ESI, one for analysis by HILIC/UPLC-MS/MS with negative ion mode ESI and one sample was reserved for backup. Proprietary software was used to match ions to an in-house library of standards for metabolite identification and quantification. Following normalization to Bradford protein concentration, log transformation and imputation of missing values, if any, with the minimum observed value for each compound, a Welch’s two-sample *t*-test was used to identify molecules that differed significantly between experimental groups.

### 4.3. Analysis of Gene Expression

A total RNA was extracted using the E.Z.N.A Total RNA kit I (OMEGA Biotek, Nocross, USA) according to the manufacturer’s protocol. Reverse transcription (RT) was done using the High Capacity Reverse Transcriptase kit (Applied Biosystems, Foster City, USA), and 1 μg of total RNA from each sample for complementary DNA synthesis. For qRT-PCR, expression levels were determined in duplicate in a LightCycler 480 real-time PCR system, using the LightCycler 480 SYBR Green I Master (Roche, Basel, Switzerland), normalized to housekeeping gene *L32*.

### 4.4. RT Profiler Array

Total RNA was extracted using the E.Z.N.A Total RNA kit I (OMEGA Biotek, Nocross, GA, USA) according to the manufacturer’s protocol and reverse-transcribed to cDNA using Reverse Transcriptase First Strand Kit (Qiagen, Hilden, Germany). The human fatty acid metabolism RT^2^ Profiler PCR Array (Qiagen, Hilden, Germany) was used to screen 84 genes in a LightCycler 480 real-time PCR system using the LightCycler 480 SYBR Green I Master (Roche, Basel, Switzerland). For data analysis, fold-changes in each gene expression were calculated using the ΔΔCt method, after normalization to a housekeeping gene (*RPLP0*) using a data analysis RT^2^ profiler platform (http://pcrdataanalysis.sabiosciences.com/pcr/arrayanalysis.php, accessed on March 2017).

### 4.5. Migration Assay

Real-time assay of cell motility was examined through the xCELLigence System (ACEA Biosciences, San Diego, CA, USA) as previously described [9]. Additional information is in Supplementary Materials and Methods.

### 4.6. Seahorse Analysis

The Seahorse analyzer XF24 (Agilent, Santa Clara, CA, USA) was used to continuously monitor OCR and ECAR. Two days prior to the experiment, 20,000 cells/well were seeded in an XF24 cell culture plate in DMEM media supplemented with 10% FBS, 25 mM glucose and 2 mM glutamine and cultivated at 37 °C in humidified atmosphere and 5% CO_2_. One day prior to the experiment, 1 mL of XF calibrator was added to each well of the XF cartridge and incubated overnight at 37 °C in humidified atmosphere and 0% CO_2_. Prior to the experiment by 30 min, cells were washed with PBS, and 625 μL of respective XF assay medium was added per well and incubated for 30 min at 37 °C in humidified atmosphere and 0% CO_2_. For the XF Cell Mito Stress analysis, XF assay medium was supplemented with 5 mM glucose and 2 mM glutamine. For the XF Glycolysis stress kit, XF assay medium was supplemented with 2 mM glutamine. After 15 min equilibration time, OCR and ECAR were accessed every 8.5 min (after 3 min mixing, 2 min wait, 3.5 min measure), and always 4 times after the addition of the respective compounds. The different compounds were added to the injection ports of the XF cartridge in 10x of final concentration and were diluted prior the experiment in XF assay medium. After the Seahorse experiment, all the cells were recovered; firstly, the medium with any floating cells was centrifuged and remaining cells were lysed using a lysis buffer (0.1 N NaOH, 0.1% SDS). Protein concentration was determined using Pierce BCA protein assay kit (Thermo Fisher Scientific, Waltham, MA, USA) for normalization. Additional information is in Supplementary Materials and Methods.

### 4.7. Lactate Production and Glucose Consumption Assays

Cells were seeded in full media in a 6-well plate (8 × 10^4^ cells in 2 mL) in triplicates. Media was replaced after 24 h. After 48 h the media was collected for analysis of L-lactate and glucose and cells were counted for normalization. The concentration of L-lactate was determined using an enzymatic reaction based on the oxidation of L-lactate to pyruvate as previously described [29]. The concentration of glucose was determined using a glucose oxidase and peroxidase method, PGO Enzymes (Sigma-Aldrich, St. Louis, MI, USA), according to manufacturer’s instructions. Additional information is in Supplementary Materials and Methods.

### 4.8. Statistical Analyses

All data represent at least three experiments and are expressed as the mean ± standard deviation (SD). Differences between groups were compared using either Student’s *t*-test or one-way ANOVA associated with the Dunnett’s test. Statistical calculation was performed using GraphPad Prism 6.01 software (GraphPad Software Inc., La Jolla, CA, USA) and statistical significance was assumed when *p* < 0.05. Metabolomic profiling results were utilized by Metabolon Inc. using a Welch’s two-sample *t*-test.

## Data Availability

Not applicable.

## Supplementary Materials

The following are available online at https://www.mdpi.com/article/10.3390/ijms22115543/s1.
