# Supplementary material for: Epithelial–Mesenchymal Transition (EMT) Induced by TGF-β in Hepatocellular Carcinoma Cells Reprograms Lipid Metabolism
Source: Int J Mol Sci. 2021 May 24;22(11):5543. doi: 10.3390/ijms22115543 (PMC8197297; doi:10.3390/ijms22115543)
Supplement: Supplementary file 1 [file ijms-22-05543-s001.zip › ijms-1203435-supplementary.pdf]

Supplementary Materials and Methods

Immunofluorescence studies

Fluorescence microscopy studies were performed as described previously [1]. Cells were fixed with methanol (100%; -20º C, 2 minutes) prior incubation with blocking solution and the addition of the primary antibodies (1:50). All primary antibodies were diluted in 1% BSA (1 hour; room temperature). After primary antibody incubation, cells were washed three times with PBS, and afterwards samples were incubated with Alexa Fluor 488- or Alexa Fluor 555- conjugated anti-mouse, for 1 hour at room temperature (1:1000) and mounted in Vectashield mounting medium. Cells were visualized in a Nikon eclipse 80i microscope with the appropriate filters. Representative images were taken with a Nikon DS-Ri1 digital camera and edited in Adobe Photoshop. Primary antibodies used: E-Cadherin, #610182 from BD Transduction Laboratories (Erembodegem, Belgium); Vimentin, #V6630 from Sigma-Aldrich (St. Louis, MO, USA); ZO-1, #61-7300 from Invitrogen (Waltham, MA, USA); Cytokeratin 18, #61028 from Progen (Heidelberg, Germany). Phalloidin (#P1951K) and Dapi (4',6-Diamidino-2-Phenylindole, Dihydrochloride, #32670) were from Sigma-Aldrich (St. Louis, MO, USA).

Analysis of gene expression

Real-time quantitative PCR reactions were performed using the following human specific primers designed by Integrated DNA Technologies (IDT):

L32	Forward: 5'-AACGTCAAGGAGCTGGAAG-3'
	Reverse: 5'-GGGTTGGTGACTCTGATGG-3'
CDH1	Forward: 5'-CCCAATACATCTCCCTTCACAG-3'
	Reverse 5'-CCACCTCTAAGGCCATCTTTG-3'
KRT18	Forward: 5'-CAGAGACTGGAGCCATTACTTC-3'
	Reverse: 5'-GCCAGCTCTGTCTCATACTTG-3'
VIM	Forward: 5'- GGAAGCCTAACTACAGCGAG -3'

	Reverse: 5’- CAGAGTCCCAGATGAGCATTG -3’
SNAI1	Forward: 5′-GCTGCAGGACTCTAATCCAGAGTT-3′
	Reverse: 5’-GACAGAGTCCCAGATGAGCATTG-3’
SNAI2	Forward: 5′- ACACATTAGAACTCACACGGG -3′
	Reverse: 5’- TGGAGAAGGTTTTGGAGCAG -3’
ZEB2	Forward: 5′-AGGCATATGGTGACGCACAA-3′
	Reverse: 5’-CTTGAACTTGCGGTTACCTGC-3’
TWIST1	Forward: 5′-CTCAGCTACGCCTTCTCG-3′
	Reverse: 5’-ACTGTCCATTTTCTCCTTCTCTG-3’
ACACA	Forward: 5’- CTGGAGGTGTATGTTCGAAGG -3’
	Reverse: 5’- TCTGTTTAGCGTAGGGATGTTC -3’
ACACB	Forward: 5’- CCCCAGACAAGTATCCCAAAG -3’
	Reverse: 5’- GGGTACTCCTGGGTCTTAAAC-3’

Targeted know-down assays

Hep3B and SNU449 cell line was transfected with a combination of 4 shRNA plasmids for TGFβRI as well as a control unspecific shRNA. After 24 hours, media was changed to complete media, and selection of transfected cells was done with puromycin (for 50 days prior to experiments).

shRNA plasmids were selected from Mission SH (Sigma-Aldrich, St Louis, USA) with following sequences:

shTβRI:

#1 –CCGGCTCATGTTGATGGTCTATATCCTCGAGGATATAGACCATCAACATGAGTTTTTG-

#2 –CCGGGAAGTTGCTGTTAAGATATTCCTCGAGGAATATCTTAACAGCAACTTCTTTTTG-

#3 –CCGGGATCATGATTACTGTCGATAACTCGAGTTATCGACAGTAATCATGATCTTTTTG-

#4 –CCGGGCTGGTCTTAACTTTAGGTAACTCGAGTTACCTAAAGTTAAGACCAGCTTTTTG-

Migration assay

Real-time assay of cell motility was examined through the xCELLigence System (ACEA Biosciences, San Diego, USA) as previously described [1]. $4x10^4$cells/well were seeded onto the top chamber of a CIM plate®, which was coated with a collagen IV solution (Sigma-Aldrich) and placed onto the Real-Time Cell Analyzer (RTCA) station (ACEA Biosciences, San Diego, USA). Continuous values were represented as Cell Index (CI), a dimensionless parameter, which reflects a relative change in measured electrical impedance, and quantified as a slope (h^{-1}) of the first 8 hours of cell migration.

Seahorse analysis

For the XF Cell Mito Stress analysis, ATP synthase inhibitor oligomycin (1μM) was injected to block proton movement and therefore oxidative phosphorylation. Consequently, carbonyl cyanide-*4*-(trifluoromethoxy) phenylhydrazone (FCCP, 0.5 μM) re-establishes proton movement by disrupting the mitochondrial membrane potential to stimulate maximal OCR and electron transport. At last, a combination of rotenone (1 μM) and antimycin (1 μM) was added to block complex I and III respectively and therefore completely block the mitochondrial respiration. The values obtained after injection of rotenone and antimycin were considered as non-mitochondrial respiration. The basal OCR was calculated as the last OCR measurement prior the addition of oligomycin minus the last OCR measurement after the addition of antimycin and rotenone. The ATP-linked OCR was calculated as the last OCR measurement prior oligomycin injection minus the last OCR measurement after the addition of oligomycin. The maximal OCR was calculated as the last OCR measurement after FCCP injection minus the last OCR measurement after the addition of antimycin and rotenone.

For the XF Glycolysis stress kit, glucose (10 mM) was injected, followed by oligomycin (1 μM), which as an ATP inhibitor permits the readout of the maximal ECAR. Finally, 2-DG (50 mM) was added to completely block glycolysis. The level of glycolysis is calculated as the last ECAR measurement after glucose injection minus the last ECAR measurement after the addition of 2-DG. The glycolytic capacity is calculated as the last ECAR measurement after oligomycin

injection minus the last ECAR measurement after 2-DG injection. The glycolytic reserve is calculated as the glycolytic capacity minus glycolysis.

Lactate production assay

The concentration of L-lactate was determined using an enzymatic reaction based on the oxidation of L-lactate to pyruvate by lactate dehydrogenase (5 mg of the enzyme (Roche, Basel, Switzerland)/ml, 550 U/mg) in the presence of NAD^+ (Sigma Aldrich) as previously described [2]. In this assay, the amount of NADH produced in the reaction is proportional to the amount of L-lactate in the samples. With this enzymatic system, D-lactate is not detected. All experiments were carried out at least 3 times in triplicates. The collected culture medium were diluted 1:20 with reaction mix [0.3 M hydrazine sulfate (Sigma Aldrich) and 0.87 M glycine (Sigma Aldrich)], pH 9.5; 2.5 M NAD^+ (Sigma Aldrich) and 0.19 M EDTA (Sigma Aldrich). Lactate dehydrogenase was added at a final concentration of 6.9 U/ml. The NADH concentration was determined as absorbance at 340 nm on a plate reader after 0 and 20 minutes after the start of the reaction. After the subtraction of absorbance at time 0, the absorbance at 20 minutes was normalization to L-lactate standards [(a serial dilution of sodium L-lactate (Sigma Aldrich)], obtained L-lactate concentrations were normalized to respective cell numbers.

Glucose consumption assay

The concentration of glucose was determined using a glucose oxidase and peroxidase method, PGO Enzymes (Sigma Aldrich) according to manufacturer’s instructions. Briefly, to prepare the PGO enzyme reaction solution, 100 ml PGO Enzymes solution (1 PGO capsule in 100 ml of distilled water) was mixed with 1.6 ml of the o-Dianisidine solution [50 mg of o-Dianisidine hydrochloride (Sigma Aldrich) in 20 ml of distilled water]. Medium samples were diluted (1/20 in distilled water) and 15µl of sample was added to 200 µl of prepared reaction mix. After 30 minutes incubation at 37°C, absorbance was read on a plate reader at 450 nm. Different concentrations of glucose solution (Sigma Aldrich) served as the standards. The glucose

consumption was calculated as the glucose concentration of medium control (no cells) minus the glucose concentration of individual samples. All experiments were carried out at least 3 times in triplicates.

References

1 Bertran, E. *et al.* Overactivation of the TGF-β pathway confers a mesenchymal-like phenotype and CXCR4-dependent migratory properties to liver tumor cells. *Hepatology* **58(6)**, 2032-2044 (2013).

2 Álvarez, Z., Hyroššová, P., Perales, J. & Alcántara, S. Neuronal Progenitor Maintenance Requires Lactate Metabolism and PEPCK-M-Directed Cataplerosis. *Cereb Cortex* **26(3)**, 1046-1058 (2016).

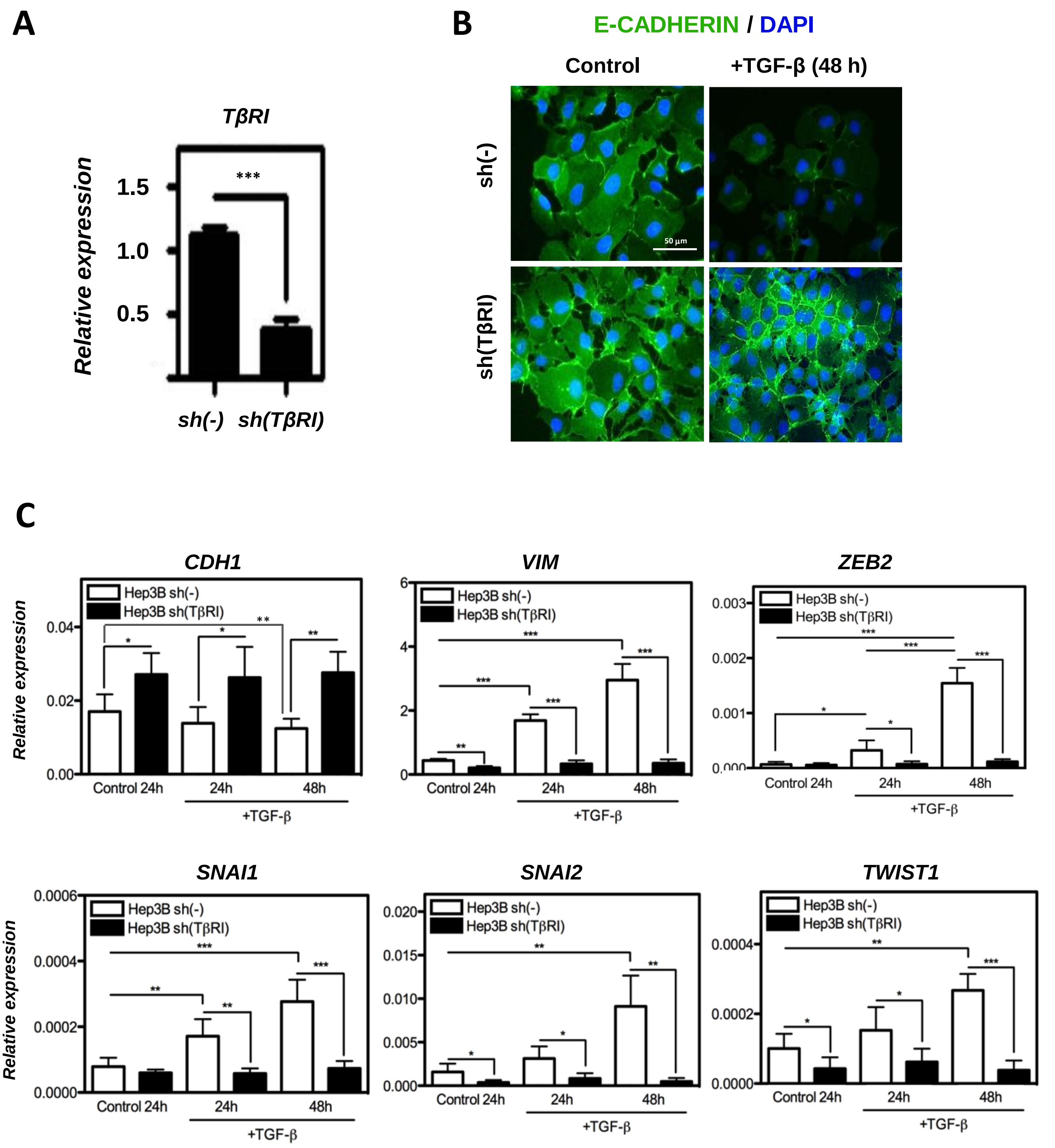

Figure S1 - Treatment of Hep3B cells with TGF-β induces Epithelial-Mesenchymal Transition (EMT), an effect that is abrogated when the TGF-β receptor I (TβRI) is silenced.

Hep3B cells, stably transfected with shcontrol (sh-) or specific TβRI shRNA (**A.**) were cultured in the absence (Control) or in the presence of TGF-β (2 ng/ml) for 24 and/or 48 h, as indicated in each graph: (**B.**): Immunofluorescence analysis of E-cadherin (green), DAPI (nuclei) in blue. Scale bar represents 50 µm. (**C.**) mRNA expression levels of EMT-related genes (E-CADHERIN: *CDH1*; VIMENTIN: *VIM*) and EMT-inducing transcription factors (*SNAI1*, *SNAI2*, *TWIST1* and *ZEB2*) were detected by qRT-PCR and normalized to housekeeping gene L32. Mean±SD (n=3). *$p<0.05$, **$p<0.01$, ***$p<0.001$, compared as indicated in the graphs.

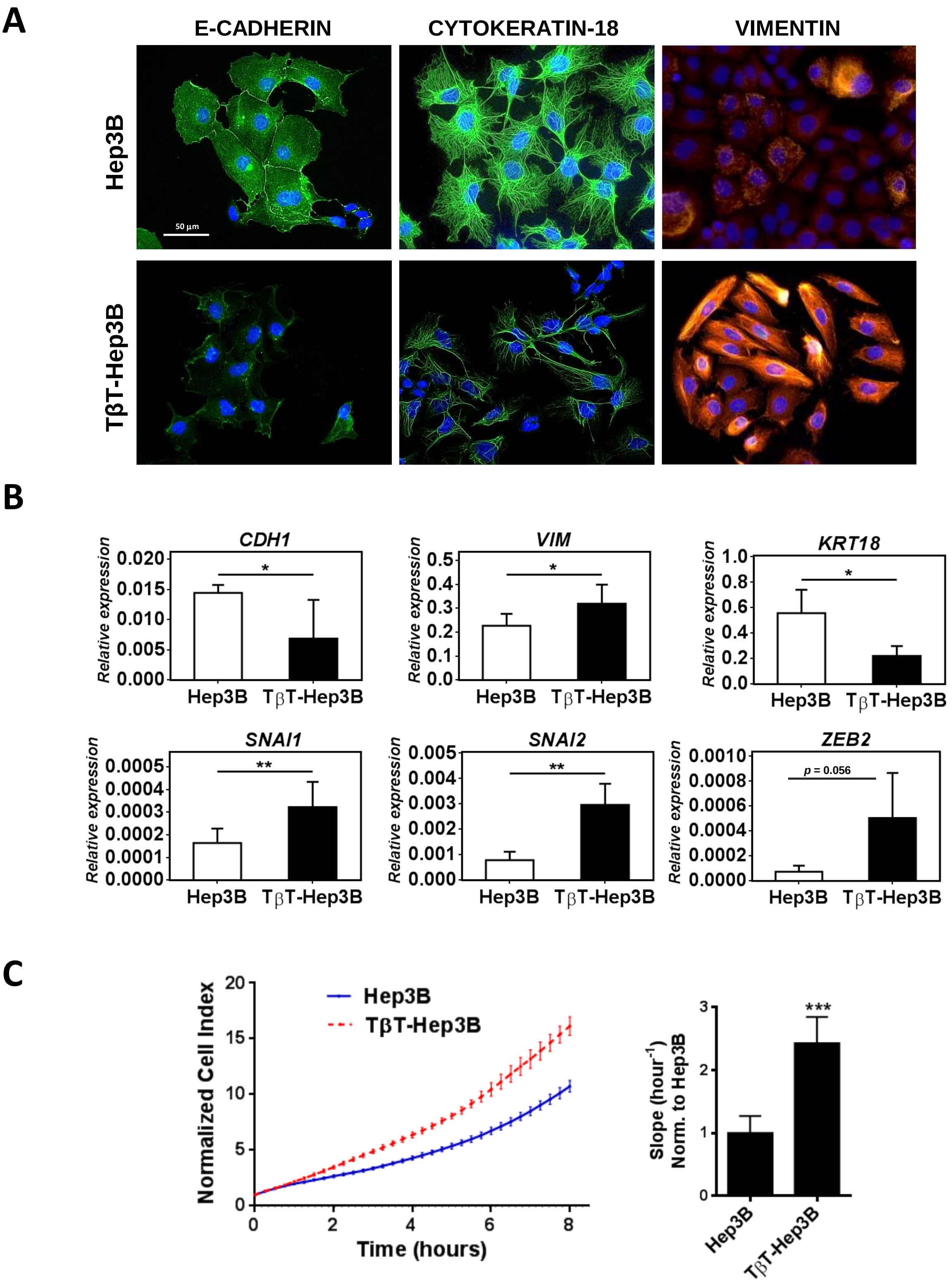

Figure S2 - Generation of TβT-Hep3B (Chronic TGF-β-treated Hep3B) cells.

Hep3B cells were treated with TGF-β (2 ng/ml) for two weeks. Media was replaced every two days. **(A.)** Immunofluorescence analysis of E-cadherin (green), Cytokeratin 18 (green) and vimentin (orange). DAPI (blue). Scale bar represents 50 µm. **(B.)** mRNA expression levels of EMT-related genes (*CDH1*, *VIM*, *KRT18*) and EMT-inducing transcription factors (*SNAI1*, *SNAI2*, *ZEB2*) analysed by qRT-PCR and normalized to housekeeping gene L32. Mean±SD (n=3). *$p<0.05$, **$p<0.01$ as compared to Hep3B. **(C.)** Analysis of cell migration by real-time assay (xCELLigence system) between Hep3B and TβT-Hep3B. Left: A representative experiment is shown. Right: Quantification as a slope (hour-1) of the first 8 hours of cell migration normalized to Hep3B. Mean±SD (n=3). ***$p<0.001$ as compared to Hep3B.

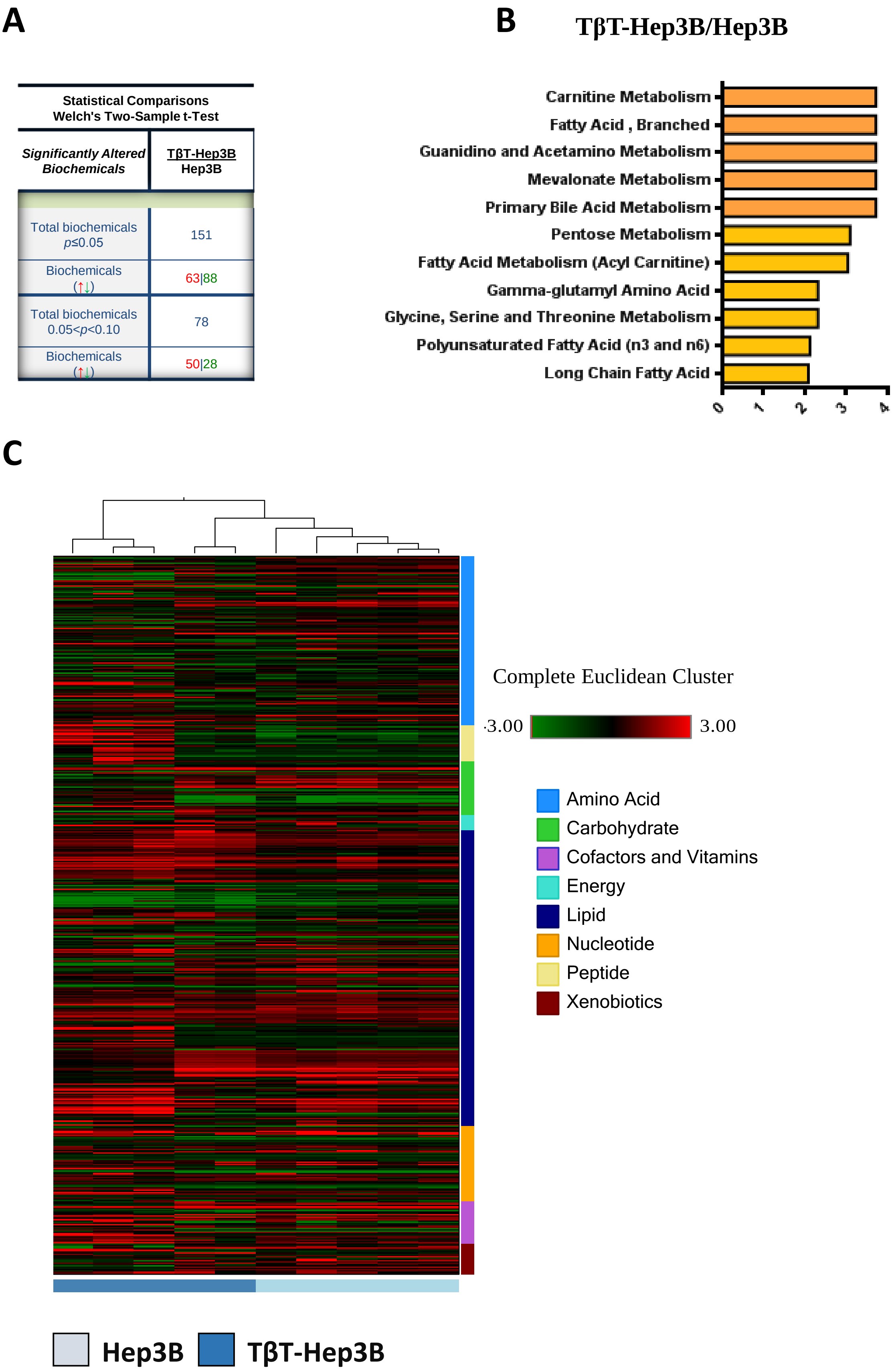

Figure S3 - Metabolomic analysis of TβT-Hep3B/Hep3B cells.
(A.) A total of 561 metabolites were detected. Welch's two-sample *t*-test was used to identify biochemicals that differed significantly ($p<0.05$) and with approaching significance ($0.05<p>0.10$) between experimental groups (n=5 for each group). **(B.)** Pathway set enrichment analysis was performed using MetaboLync^{TM} platform and followed formula: # of significant metabolites in pathway (k) / total # of detected metabolites in pathway (m)) / [total # of significant metabolites (n) / total # of detected metabolites(N)] (k/m)/(n/N). **(C.)** Hierarchical clustering was performed using Euclidean distance method by Metabolon Inc.

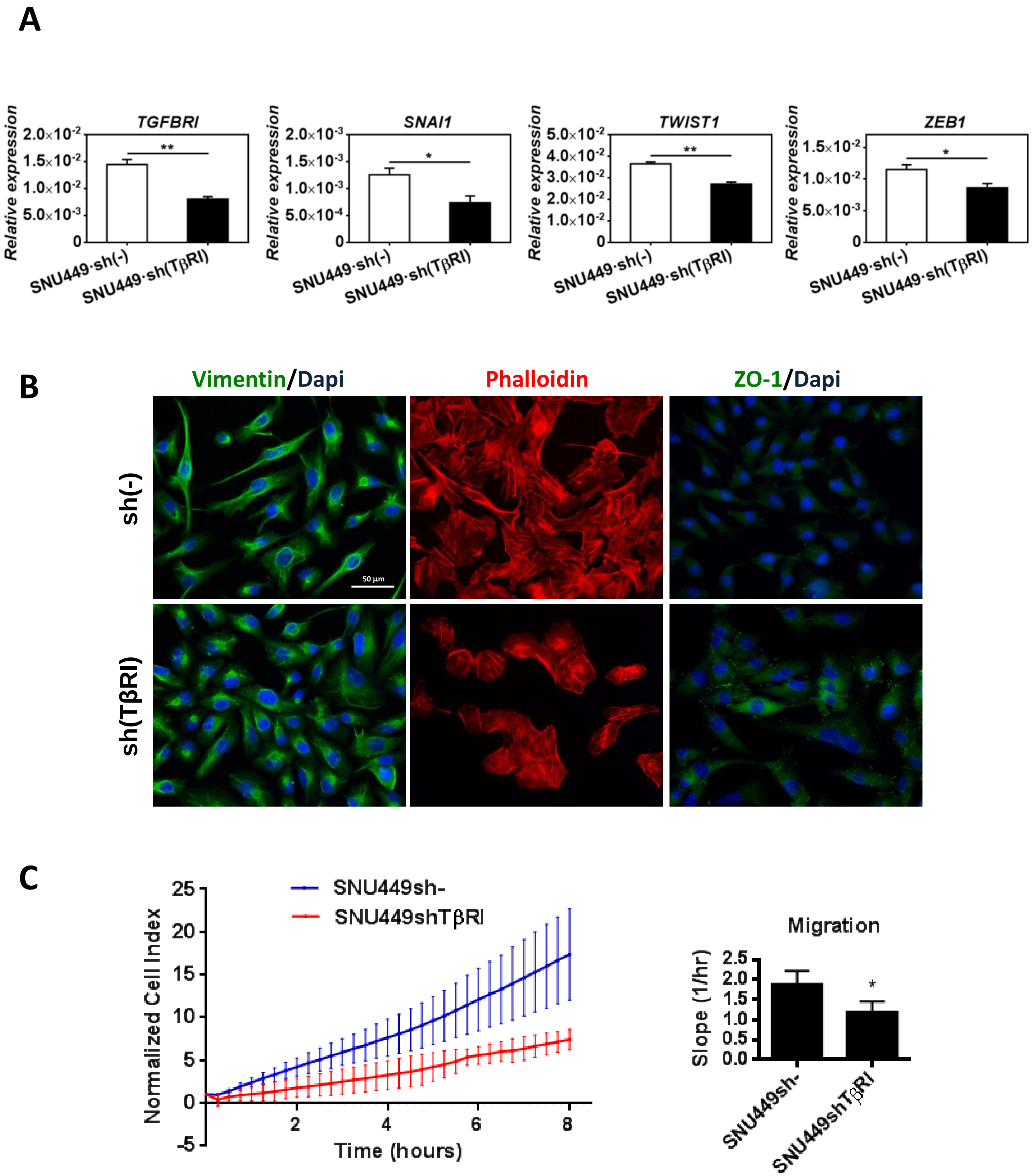

Figure S4 – Effect of silencing the TGF-β receptor I (TβRI) in the mesenchymal HCC cell line SNU449.
SNU449 cells, control (sh-) or stably silenced with specific TβRI shRNA (**A.**) mRNA expression levels of *TGFBRI* and the EMT-inducing transcription factors (*SNAI1*, *TWIST1* and *ZEB1*) were detected by qRT-PCR and normalized to housekeeping gene L32. (**B.**): Immunofluorescence analysis of Vimentin and ZO1 (green) and staining analysis of F-Actin with phalloidin (red), DAPI (nuclei) in blue. Scale bar represents 50 µm. (**C.**) Analysis of cell migration by real-time assay (xCELLigence system) between Hep3B and TβT-Hep3B. Left: A representative experiment is shown. Right: Quantification as a slope (hour-1) of the first 8 hours of cell migration. In A. and C.: Mean±SD (n=3). *p<0.05, **p<0.01, ***p<0.001, compared as the graph indicates.

Table S1.

List of dectected metabolites in TβT-Hep3B compared to Hep3B

Sub Pathway	Biochemical Name	TβT-Hep3B / Hep3B	p value
Short Chain Fatty Acid	valerate	1.40	0.2504
Medium Chain Fatty Acid	caproate (6:0)	1.52	0.0839
	caprylate (8:0)	1.43	0.1645
	laurate (12:0)	1.10	0.9904
	5-dodecenoate (12:1n7)	1.15	0.6963
Long Chain Fatty Acid	myristate (14:0)	1.32	0.1339
	myristoleate (14:1n5)	1.24	0.5979
	pentadecanoate (15:0)	**1.32**	0.0179
	palmitate (16:0)	1.15	0.2517
	palmitoleate (16:1n7)	1.22	0.3616
	margarate (17:0)	**1.76**	0.0061
	10-heptadecenoate (17:1n7)	**1.51**	0.0031
	stearate (18:0)	**1.43**	0.0066
	nonadecanoate (19:0)	1.26	0.1043
	10-nonadecenoate (19:1n9)	**3.05**	0.0003
	arachidate (20:0)	1.23	0.1794
	eicosenoate (20:1)	**2.30**	0.0001
	tricosenoate (23:1)	**2.68**	0.0039
	behenate (22:0)	1.96	0.1175
	erucate (22:1n9)	**1.76**	0.0007
	oleate/vaccenate (18:1)	**1.72**	0.0005
Polyunsaturated Fatty Acid (n3 and n6)	stearidonate (18:4n3)	1.51	0.0908
	eicosapentaenoate (EPA; 20:5n3)	0.80	0.2494
	docosapentaenoate (n3 DPA; 22:5n3)	**2.78**	0.0081
	docosahexaenoate (DHA; 22:6n3)	0.96	0.7408
	docosatrienoate (22:3n3)	**2.32**	0.0079
	linoleate (18:2n6)	**1.52**	0.0027
	linolenate [alpha or gamma; (18:3n3 or 6)]	1.30	0.1831
	dihomo-linolenate (20:3n3 or n6)	**1.92**	0.0429
	arachidonate (20:4n6)	0.95	0.8391
	adrenate (22:4n6)	6.13	0.1290
	docosapentaenoate (n6 DPA; 22:5n6)	**2.18**	0.0284
	docosadienoate (22:2n6)	**2.57**	0.0000
	dihomo-linoleate (20:2n6)	**3.09**	0.0007
	mead acid (20:3n9)	**1.91**	0.0053
Fatty Acid, Branched	13-methylmyristate	**1.73**	0.0005
	15-methylpalmitate	**1.67**	0.0002
	17-methylstearate	**1.94**	0.0071
Fatty Acid, Dicarboxylate	2-hydroxyglutarate	**0.31**	0.0051
	2-hydroxyadipate	0.56	0.1234
	maleate	0.80	0.2015
	pimelate (heptanedioate)	**2.30**	0.0410
Fatty Acid Metabolism	acetyl CoA	1.00	---
Fatty Acid Metabolism (also	butyrylcarnitine	0.13	0.0729
	butyrylglycine	1.20	0.3737

BCAA Metabolism)	propionylcarnitine	0.88	0.3739
	methylmalonate (MMA)	**8.03**	0.0221
Fatty Acid Metabolism(Acyl Glycine)	N-palmitoylglycine	1.00	---
Fatty Acid Metabolism(Acyl Carnitine)	acetylcarnitine	**0.37**	0.0133
	3-hydroxybutyrylcarnitine (1)	**0.29**	0.0303
	3-hydroxybutyrylcarnitine (2)	**0.20**	0.0289
	hexanoylcarnitine	**0.12**	0.0016
	myristoylcarnitine	**0.38**	0.0098
	palmitoylcarnitine	**0.30**	0.0182
	palmitoleoylcarnitine	**0.25**	0.0003
	stearoylcarnitine	0.78	0.2586
	linoleoylcarnitine	**0.64**	0.0292
	oleoylcarnitine	**0.18**	0.0207
	myristoleoylcarnitine	1.36	0.3272
Carnitine Metabolism	deoxycarnitine	**0.53**	0.0015
	carnitine	**0.63**	0.0197
Fatty Acid, Monohydroxy	4-hydroxybutyrate (GHB)	4.86	0.0964
	2-hydroxypalmitate	1.54	0.1974
	2-hydroxystearate	1.66	0.1807
	3-hydroxyhexanoate	**1.67**	0.0036
	3-hydroxyoctanoate	**1.52**	0.0253
	3-hydroxydecanoate	**1.63**	0.0076
	3-hydroxylaurate	1.10	0.6897
	3-hydroxypalmitate	0.89	0.2564
	13-HODE + 9-HODE	1.05	0.4504
	3-hydroxystearate	0.59	0.3031
Inositol Metabolism	myo-inositol	**0.55**	0.0003
	inositol 1-phosphate (I1P)	0.66	0.0887
	choline	1.03	0.7164
	choline phosphate	0.79	0.1359
	cytidine 5'-diphosphocholine	1.20	0.7929
	glycerophosphorylcholine (GPC)	5.75	0.1725
	phosphoethanolamine	**0.33**	0.0054
	cytidine-5'-diphosphoethanolamine	**0.45**	0.0326
	glycerophosphoethanolamine	0.90	0.7911
	trimethylamine N-oxide	0.93	0.4093
	glycerophosphoinositol	0.71	0.6835
	1,2-dipalmitoyl-GPC (16:0/16:0)	0.59	0.0653
	1-palmitoyl-2-oleoyl-GPC (16:0/18:1)	1.09	0.1505
	1-palmitoyl-2-linoleoyl-GPC (16:0/18:2)	**0.75**	0.0309
	1-stearoyl-2-arachidonoyl-GPC (18:0/20:4)	2.29	0.0942
	1-stearoyl-2-oleoyl-GPC (18:0/18:1)	2.72	0.0566
	1-stearoyl-2-oleoyl-GPI (18:0/18:1)	2.06	0.0733
	1,2-dioleoyl-GPC (18:1/18:1)	2.48	0.0535
	1-palmitoyl-2-arachidonoyl-GPC (16:0/20:4)	0.82	0.2087
	1-stearoyl-2-linoleoyl-GPC (18:0/18:2)	1.85	0.0992
	1-palmitoleoyl-2-oleoyl-GPC (16:1/18:1)	1.28	0.2020
	1-palmitoyl-2-palmitoleoyl-GPC (16:0/16:1)	**0.60**	0.0306

Phospholipid Metabolism	1-stearoyl-2-arachidonoyl-GPI (18:0/20:4)	**0.69**	0.0363
	1-oleoyl-2-linoleoyl-GPC (18:1/18:2)	1.20	0.0802
	1-palmitoyl-2-arachidonoyl-GPI (16:0/20:4)	**0.51**	0.0414
	1-palmitoyl-2-oleoyl-GPG (16:0/18:1)	0.76	0.2119
	1-palmitoyl-2-oleoyl-GPE (16:0/18:1)	**0.72**	0.0496
	1-stearoyl-2-arachidonoyl-GPE (18:0/20:4)	0.83	0.1658
	1-stearoyl-2-oleoyl-GPE (18:0/18:1)	**2.65**	0.0402
	1-palmitoyl-2-arachidonoyl-GPE (16:0/20:4)	**0.44**	0.0306
	1-palmitoyl-2-linoleoyl-GPE (16:0/18:2)	**0.43**	0.0273
	1-stearoyl-2-linoleoyl-GPE (18:0/18:2)	0.79	0.0857
	1-palmitoyl-2-stearoyl-GPC (16:0/18:0)	**0.60**	0.0356
	1,2-dioleoyl-GPE (18:1/18:1)	**1.67**	0.0026
	1-palmitoyl-2-oleoyl-GPI (16:0/18:1)	**3.13**	0.0170
	1,2-dioleoyl-GPS (18:1/18:1)	2.00	0.0503
	1-palmitoyl-2-alpha-linolenoyl-GPC (16:0/18:3n3)	0.77	0.1573
	1-palmitoleoyl-2-linoleoyl-GPC (16:1/18:2)	**0.59**	0.0043
	1-palmitoleoyl-2-linolenoyl-GPC (16:1/18:3)	**0.40**	0.0415
	1,2-dilinoleoyl-GPC (18:2/18:2)	1.06	0.5679
	1-oleoyl-2-linoleoyl-GPE (18:1/18:2)	0.67	0.0552
	1,2-distearoyl-GPC (18:0/18:0)	**0.42**	0.0341
	1-palmitoyl-2-oleoyl-GPS (16:0/18:1)	0.68	0.0579
	1-stearoyl-2-linoleoyl-GPS (18:0/18:2)	1.29	0.1616
	oleoylcholine	1.32	0.8961
	palmitoloelycholine	0.89	0.7288
	1-palmitoyl-2-gamma-linolenoyl-GPC (16:0/18:3n6)	**0.56**	0.0026
Phosphatidylserine (PS)	1-stearoyl-2-arachidonoyl-GPS (18:0/20:4)	0.70	0.1537
	1-stearoyl-2-oleoyl-GPS (18:0/18:1)	3.81	0.0551
Lysolipid	1-palmitoyl-GPC (16:0)	**0.60**	0.0275
	2-palmitoyl-GPC (16:0)	0.84	0.2420
	1-palmitoleoyl-GPC (16:1)	**0.48**	0.0038
	2-palmitoleoyl-GPC (16:1)	1.19	0.2436
	1-stearoyl-GPC (18:0)	1.10	0.7946
	1-oleoyl-GPC (18:1)	1.05	0.6838
	1-linoleoyl-GPC (18:2)	0.90	0.4276
	1-arachidonoyl-GPC (20:4n6)	1.60	0.0902
	1-lignoceroyl-GPC (24:0)	8.62	0.0804
	1-palmitoyl-GPE (16:0)	**0.30**	0.0224
	1-stearoyl-GPE (18:0)	**0.52**	0.0035
	2-stearoyl-GPE (18:0)	**0.47**	0.0080
	1-oleoyl-GPE (18:1)	0.74	0.0945
	1-linoleoyl-GPE (18:2)	0.84	0.3231
	1-arachidonoyl-GPE (20:4n6)	0.98	0.8755
	1-palmitoyl-GPI (16:0)	1.13	0.6493
	1-stearoyl-GPI (18:0)	1.41	0.1793
	1-oleoyl-GPI (18:1)	**2.58**	0.0112
	1-arachidonoyl-GPI (20:4)	1.48	0.0909
	1-stearoyl-GPS (18:0)	**0.58**	0.0135

	1-oleoyl-GPS (18:1)	2.07	0.0824
	1-palmitoyl-GPG (16:0)	0.89	0.5518
	1-palmitoyl-GPS (16:0)	**0.34**	0.0342
	1-stearoyl-GPG (18:0)	1.00	---
	1-oleoyl-GPG (18:1)	1.13	0.6704
	1-linoleoyl-GPG (18:2)	1.10	0.8341
Plasmalogen	1-(1-enyl-palmitoyl)-2-oleoyl-GPE (P-16:0/18:1)	12.66	0.0552
	1-(1-enyl-palmitoyl)-2-linoleoyl-GPE (P-16:0/18:2)	26.21	0.0890
	1-(1-enyl-palmitoyl)-2-palmitoyl-GPC (P-16:0/16:0)	**0.75**	0.0187
	1-(1-enyl-palmitoyl)-2-palmitoleoyl-GPC (P-16:0/16:1)	1.43	0.7890
	1-(1-enyl-palmitoyl)-2-arachidonoyl-GPE (P-16:0/20:4)	0.85	0.3651
	1-(1-enyl-palmitoyl)-2-oleoyl-GPC (P-16:0/18:1)	1.95	0.2768
	1-(1-enyl-stearoyl)-2-oleoyl-GPE (P-18:0/18:1)	**22.43**	0.0208
	1-(1-enyl-stearoyl)-2-linoleoyl-GPE (P-18:0/18:2)	**53.55**	0.0153
	1-(1-enyl-palmitoyl)-2-arachidonoyl-GPC (P-16:0/20:4)	1.00	---
	1-(1-enyl-stearoyl)-2-arachidonoyl-GPE (P-18:0/20:4)	0.87	0.2479
Lysoplasmalogen	1-(1-enyl-palmitoyl)-GPC (P-16:0)	1.38	0.1960
	1-(1-enyl-palmitoyl)-GPE (P-16:0)	7.43	0.0886
	1-(1-enyl-oleoyl)-GPE (P-18:1)	5.80	0.1412
	1-(1-enyl-stearoyl)-GPE (P-18:0)	5.03	0.1057
	1-(1-enyl-oleoyl)-2-oleoyl-GPE (P-18:1/18:1)	**59.98**	0.0123
Glycerolipid Metabolism	glycerol	**4.35**	0.0006
	glycerol 3-phosphate	0.63	0.2377
	glycerophosphoglycerol	1.36	0.5881
Monoacylglycerol	1-myristoylglycerol (14:0)	1.14	0.6593
	2-myristoylglycerol (14:0)	1.34	0.8906
	1-myristoleoylglycerol (14:1)	0.66	0.1597
	1-pentadecanoylglycerol (15:0)	1.00	0.6381
	1-palmitoylglycerol (16:0)	1.04	0.5856
	2-palmitoylglycerol (16:0)	1.27	0.9940
	1-margaroylglycerol (17:0)	1.42	0.4550
	1-oleoylglycerol (18:1)	1.23	0.9220
	2-oleoylglycerol (18:1)	1.32	0.7477
	1-linoleoylglycerol (18:2)	0.77	0.2275
	2-linoleoylglycerol (18:2)	0.81	0.2623
	1-linolenoylglycerol (18:3)	0.68	0.1573
	1-arachidonylglycerol (20:4)	0.78	0.2361
	2-arachidonoylglycerol (20:4)	0.82	0.3202
	1-docosahexaenoylglycerol (22:6)	0.66	0.1383
	1-dihomo-linolenylglycerol (20:3)	0.78	0.2203
	2-docosahexaenoylglycerol (22:6)	0.72	0.1949
	1-palmitoleoylglycerol (16:1)	0.75	0.1916
	2-palmitoleoylglycerol (16:1)	0.79	0.2169
	1-eicosapentaenoylglycerol (20:5)	0.62	0.1297
	2-eicosapentaenoylglycerol (20:5)	0.65	0.1938

Diacylglycerol	palmitoleoyl-oleoyl-glycerol (16:1/18:1) [1]	**0.44**	0.0492
	oleoyl-linoleoyl-glycerol (18:1/18:2) [2]	1.08	0.5366
	palmitoleoyl-oleoyl-glycerol (16:1/18:1) [2]	0.75	0.2098
	palmitoyl-linoleoyl-glycerol (16:0/18:2) [1]	**0.34**	0.0310
	palmitoyl-linoleoyl-glycerol (16:0/18:2) [2]	0.76	0.2265
Sphingolipid Metabolism	N-palmitoyl-sphinganine (d18:0/16:0)	1.65	0.1425
	sphinganine	**2.01**	0.0003
	phytosphingosine	1.31	0.8474
	palmitoyl sphingomyelin (d18:1/16:0)	1.46	0.1420
	stearoyl sphingomyelin (d18:1/18:0)	1.47	0.3705
	sphingomyelin (d18:1/18:1, d18:2/18:0)	1.43	0.2397
	sphingosine	**1.90**	0.0012
	N-palmitoyl-sphingosine (d18:1/16:0)	**2.50**	0.0495
	sphingomyelin (d18:1/14:0, d16:1/16:0)	1.06	0.2318
	sphingomyelin (d18:2/14:0, d18:1/14:1)	1.54	0.0557
	sphingomyelin (d18:1/24:1, d18:2/24:0)	1.36	0.7458
	sphingomyelin (d18:2/16:0, d18:1/16:1)	1.19	0.4829
	sphingomyelin (d18:1/20:1, d18:2/20:0)	0.78	0.1103
	behenoyl sphingomyelin (d18:1/22:0)	0.89	0.4427
	sphingomyelin (d18:1/22:1, d18:2/22:0, d16:1/24:1)	0.99	0.7914
	sphingomyelin (d18:1/20:0, d16:1/22:0)	**0.74**	0.0405
	palmitoyl dihydrosphingomyelin (d18:0/16:0)	**0.70**	0.0452
	sphingomyelin (d18:1/15:0, d16:1/17:0)	1.01	0.8567
	sphingomyelin (d18:1/21:0, d17:1/22:0, d16:1/23:0)	1.09	0.6043
	sphingomyelin (d18:2/23:0, d18:1/23:1, d17:1/24:1)	1.46	0.3541
	sphingomyelin (d18:2/24:1, d18:1/24:2)	1.59	0.5426
	tricosanoyl sphingomyelin (d18:1/23:0)	1.02	0.9753
	sphingomyelin (d18:1/17:0, d17:1/18:0, d19:1/16:0)	3.88	0.0901
	glycosyl-N-stearoyl-sphingosine	**11.31**	0.0459
	glycosyl-N-palmitoyl-sphingosine	**4.08**	0.0159
	lactosyl-N-palmitoyl-sphingosine	**4.27**	0.0088
Mevalonate Metabolism	3-hydroxy-3-methylglutarate	**0.38**	0.0007
Glycolysis, Gluconeogenesis, and Pyruvate Metabolism	glucose	0.94	0.5541
	glucose 6-phosphate	4.49	0.0673
	fructose-6-phosphate	**2.22**	0.0323
	Isobar: fructose 1,6-diphosphate, glucose 1,6-diphosphate, myo-inositol 1,4 or 1,3-diphosphate	0.56	0.1159
	dihydroxyacetone phosphate (DHAP)	0.77	0.2202
	3-phosphoglycerate	0.99	0.6846
	phosphoenolpyruvate (PEP)	0.62	0.0736
	pyruvate	1.12	0.6783
	lactate	0.81	0.0830
	glycerate	2.02	0.1113
	citrate	0.50	0.0629
	aconitate [cis or trans]	1.00	0.7524

TCA Cycle	isocitrate	1.16	0.3739
	alpha-ketoglutarate	0.68	0.1351
	succinylcarnitine	1.09	0.7225
	succinate	0.46	0.0668
	fumarate	0.88	0.3338
	malate	0.89	0.4420
	itaconate	**40.08**	0.0329
	2-methylcitrate/homocitrate	1.10	0.3451
Oxidative Phosphorylation	acetylphosphate	0.70	0.1904
	phosphate	1.19	0.1575
Glutamate Metabolism	glutamate	**0.74**	0.0234
	glutamine	0.97	0.6107
	N-acetylglutamate	**0.24**	0.0157
	N-acetylglutamine	**0.43**	0.0411
	N-acetyl-aspartyl-glutamate (NAAG)	**0.36**	0.0391
	gamma-aminobutyrate (GABA)	1.58	0.3181
	4-hydroxyglutamate	**8.55**	0.0002
	glutamate, gamma-methyl ester	2.21	0.1721
	pyroglutamine*	0.82	0.2281
	gamma-carboxyglutamate	1.49	0.1142
	S-1-pyrroline-5-carboxylate	1.82	0.2485

List of detected metabolites. Statistical comparison of fold-changes between groups (TβT-Hep3B/Hep3B) was performed by Welch's two-sample t-test. $p<0.05$ was considered significant. Values <1 indicate lower levels (green shading colour when significant) and values >1 indicate higher levels (red shading colour when significant) as compared to Hep3B.

Table S2.

List of analyzed genes in TβT-Hep3B and compared to Hep3B

Sub pathway	Symbol	GenBank	Description	TβT-Hep3B / Hep3B	p-value
Acetyl-CoA transferases	***ACAA1***	NM_001607	Acetyl-CoA acyltransferase 1	1.49	0.0554
	ACAA2	NM_006111	Acetyl-CoA acyltransferase 2	0.86	0.2428
	ACAT1	NM_000019	Acetyl-CoA acetyltransferase 1	0.98	0.8353
	ACAT2	NM_005891	Acetyl-CoA acetyltransferase 2	**1.91**	0.0015
Acyl-CoA dehydrogenases	***ACAD9***	NM_014049	Acyl-CoA dehydrogenase family, member 9	1.18	0.1576
	ACAD10	NM_025247	Acyl-CoA dehydrogenase family, member 10	1.36	0.1942
	ACAD11	NM_032169	Acyl-CoA dehydrogenase family, member 11	**1.59**	0.0127
	ACADL	NM_001608	Acyl-CoA dehydrogenase, long chain	0.01	0.0883
	ACADM	NM_000016	Acyl-CoA dehydrogenase, C-4 to C-12 straight chain	1.31	0.4198
	ACADS	NM_000017	Acyl-CoA dehydrogenase, C-2 to C-3 short chain	1.23	0.3524
	ACADSB	NM_001609	Acyl-CoA dehydrogenase, short/branched chain	1.03	0.7327
	ACADVL	NM_000018	Acyl-CoA dehydrogenase, very long chain	**1.80**	0.0110
	EHHADH	NM_001966	Enoyl-CoA, hydratase/3-hydroxyacyl CoA dehydrogenase	**1.69**	0.0444
	GCDH	NM_000159	Glutaryl-CoA dehydrogenase	1.06	0.7648
Acyl-CoA oxidases	***ACOX1***	NM_004035	Acyl-CoA oxidase 1, palmitoyl	0.85	0.1596
	ACOX2	NM_003500	Acyl-CoA oxidase 2, branched chain	0.79	0.5230
	ACOX3	NM_003501	Acyl-CoA oxidase 3, pristanoyl	1.16	0.3687
Acyl-CoA synthetases	***ACACA***	NM_198834	Acetyl-CoA Carboxylase Alpha	1.32	0.7337
	ACACB	NM_001093	Acetyl-CoA Carboxylase Beta	2.31	0.3639
	ACLY	NM_001096	ATP citrate lyase	**1.36**	0.0456
	ACSBG1	NM_015162	Acyl-CoA synthetase bubblegum family member 1	N/A	N/A
	ACSBG2	NM_030924	Acyl-CoA synthetase bubblegum family member 2	N/A	N/A
	ACSL1	NM_001995	Acyl-CoA synthetase long-chain family member 1	**0.38**	0.0025
	ACSL3	NM_004457	Acyl-CoA synthetase long-chain family member 3	**2.42**	0.0003
	ACSL4	NM_004458	Acyl-CoA synthetase long-chain family member 4	1.26	0.1378
	ACSL5	NM_016234	Acyl-CoA synthetase long-chain family member 5	**5.60**	0.0008
	ACSL6	NM_001009185	Acyl-CoA synthetase long-chain family member 6	**2.23**	0.0168
	ACSM3	NM_005622	Acyl-CoA synthetase medium-chain family member 3	3.05	0.1646
	ACSM4	NM_001080454	Acyl-CoA synthetase medium-chain family member 4	N/A	N/A
	ACSM5	NM_017888	Acyl-CoA synthetase medium-chain family member 5	N/A	N/A
Acyl-CoA thioesterases	***ACOT1***	NM_001037161	Acyl-CoA thioesterase 1	1.37	0.1767
	ACOT6	NM_001037162	Acyl-CoA thioesterase 6	N/A	N/A
	ACOT7	NM_181866	Acyl-CoA thioesterase 7	0.94	0.6966
	ACOT8	NM_005469	Acyl-CoA thioesterase 8	**1.67**	0.0109
	ACOT9	NM_001033583	Acyl-CoA thioesterase 9	0.97	0.7930
	ACOT12	NM_130767	Acyl-CoA thioesterase 12	N/A	N/A
	CPT1A	NM_001876	Carnitine palmitoyltransferase 1A (liver)	0.82	0.1909
	CPT1B	NM_004377	Carnitine palmitoyltransferase 1B (muscle)	N/A	N/A
	CPT1C	NM_152359	Carnitine palmitoyltransferase 1C	N/A	N/A

Fatty acid transport	***CPT2***	NM_000098	Carnitine palmitoyltransferase 2	1.06	0.8746
	CRAT	NM_000755	Carnitine O-acetyltransferase	2.39	0.0025
	CROT	NM_021151	Carnitine O-octanoyltransferase	1.16	0.1913
	FABP1	NM_001443	Fatty acid binding protein 1, liver	4.68	0.0002
	FABP2	NM_000134	Fatty acid binding protein 2, intestinal	N/A	N/A
	FABP3	NM_004102	Fatty acid binding protein 3, muscle and heart (mammary-derived growth	2.01	0.0092
	FABP4	NM_001442	Fatty acid binding protein 4, adipocyte	N/A	N/A
	FABP5	NM_001444	Fatty acid binding protein 5 (psoriasis-associated)	1.29	0.2563
	FABP6	NM_001445	Fatty acid binding protein 6, ileal	1.41	0.1823
	FABP7	NM_001446	Fatty acid binding protein 7, brain	N/A	N/A
	SLC27A1	NM_198580	Solute carrier family 27 (fatty acid transporter), member 1	3.82	0.0289
	SLC27A2	NM_003645	Solute carrier family 27 (fatty acid transporter), member 2	0.99	0.9481
	SLC27A3	NM_024330	Solute carrier family 27 (fatty acid transporter), member 3	1.63	0.1217
	SLC27A4	NM_005094	Solute carrier family 27 (fatty acid transporter), member 4	1.43	0.0163
	SLC27A5	NM_012254	Solute carrier family 27 (fatty acid transporter), member 5	1.76	0.0058
	SLC27A6	NM_014031	Solute carrier family 27 (fatty acid transporter), member 6	N/A	N/A
Biosynthesis regulation	***FASN***	NM_004104	Fatty acid synthase	1.14	0.5467
	PPAR α	NM_001001928	Peroxisome proliferator-activated receptor alpha	1.67	0.3758
	PPAR γ	NM_138712.3	Peroxisome proliferator-activated receptor gamma	2.65	0.0815
	PPAR δ	NM_006238.4	Peroxisome proliferator-activated receptor delta	1.40	0.4283
	PRKAA1	NM_006251	Protein kinase, AMP-activated, alpha 1 catalytic subunit	1.21	0.0501
	PRKAA2	NM_006252	Protein kinase, AMP-activated, alpha 2 catalytic subunit	0.76	0.0277
	PRKAB1	NM_006253	Protein kinase, AMP-activated, beta 1 non-catalytic subunit	1.47	0.0379
	PRKAB2	NM_005399	Protein kinase, AMP-activated, beta 2 non-catalytic subunit	0.81	0.0186
	PRKACA	NM_002730	Protein kinase, cAMP-dependent, catalytic, alpha	1.12	0.5596
	PRKACB	NM_182948	Protein kinase, cAMP-dependent, catalytic, beta	1.01	0.8986
	PRKAG1	NM_002733	Protein kinase, AMP-activated, gamma 1 non-catalytic subunit	1.33	0.0048
	PRKAG2	NM_016203	Protein kinase, AMP-activated, gamma 2 non-catalytic subunit	2.36	0.0004
	PRKAG3	NM_017431	Protein kinase, AMP-activated, gamma 3 non-catalytic subunit	N/A	N/A
Ketogenesis	***BDH1***	NM_004051	3-hydroxybutyrate dehydrogenase, type 1	1.01	0.8478
	BDH2	NM_020139	3-hydroxybutyrate dehydrogenase, type 2	0.95	0.6457
	HMGCL	NM_000191	3-hydroxymethyl-3-methylglutaryl-CoA lyase	1.65	0.0080
	HMGCS1	NM_002130	3-hydroxy-3-methylglutaryl-CoA synthase 1 (soluble)	2.13	0.0148
	HMGCS2	NM_005518	3-hydroxy-3-methylglutaryl-CoA synthase 2 (mitochondrial)	N/A	N/A
	OXCT2	NM_022120	3-oxoacid CoA transferase 2	0.68	0.0800
Triacylglycerol	***GK***	NM_000167	Glycerol kinase	0.85	0.6554
	GK2	NM_033214	Glycerol kinase 2	N/A	N/A
	GPD1	NM_005276	Glycerol-3-phosphate dehydrogenase 1 (soluble)	1.65	0.0418

[illegible] metabolism	***GPD2***	NM_000408	Glycerol-3-phosphate dehydrogenase 2 (mitochondrial)	1.17	0.1327
	LIPE	NM_005357	Lipase, hormone-sensitive	2.61	0.0719
	LPL	NM_000237	Lipoprotein lipase	N/A	N/A
Others	***ALDH2***	NM_000690	Aldehyde dehydrogenase 2 family (mitochondrial)	1.29	0.3847
	DECR1	NM_001359	2,4-dienoyl CoA reductase 1, mitochondrial	**0.71**	0.0231
	DECR2	NM_020664	2,4-dienoyl CoA reductase 2, peroxisomal	1.03	0.9130
	ECHS1	NM_004092	Enoyl CoA hydratase, short chain, 1, mitochondrial	1.14	0.2265
	ECI2	NM_006117	Enoyl-CoA delta isomerase 2	0.82	0.0781
	HADHA	NM_000182	Hydroxyacyl-CoA dehydrogenase/3-ketoacyl-CoA thiolase/enoyl-CoA hydratase	1.31	0.0945
	MCEE	NM_032601	Methylmalonyl CoA epimerase	**1.60**	0.0005
	MUT	NM_000255	Methylmalonyl CoA mutase	1.37	0.0949
	PECR	NM_018441	Peroxisomal trans-2-enoyl-CoA reductase	**1.61**	0.0150
	PPA1	NM_021129	Pyrophosphatase (inorganic) 1	1.04	0.6164

Sub Pathway	Symbol	GenBank	Description	TβT-Hep3B / Hep3B	p-value
Glycolysis	***ALDOA***	NM_000034	Aldolase A, fructose-bisphosphate	1.54	0.3746
	ALDOB	NM_000035	Aldolase B, fructose-bisphosphate	N/A	N/A
	ALDOC	NM_005165	Aldolase C, fructose-bisphosphate	**1.49**	0.0262
	BPGM	NM_001724	2,3-bisphosphoglycerate mutase	1.59	0.0643
	ENO1	NM_001428	Enolase 1, (alpha)	0.93	0.7141
	ENO2	NM_001975	Enolase 2 (gamma, neuronal)	0.50	0.1153
	ENO3	NM_001976	Enolase 3 (beta, muscle)	0.69	0.2976
	GALM	NM_138801	Galactose mutarotase (aldose 1-epimerase)	0.84	0.4465
	GCK	NM_000162	Glucokinase (hexokinase 4)	N/A	N/A
	GPI	NM_000175	Glucose-6-phosphate isomerase	1.55	0.3617
	HK2	NM_000189	Hexokinase 2	1.81	0.6340
	HK3	NM_002115	Hexokinase 3 (white cell)	N/A	N/A
	LDHA	NM_005566	Lactate dehydrogenase A	1.22	0.2212
	LDHB	NM_002300	Lactate dehydrogenase B	N/A	N/A
	PFKL	NM_002626	Phosphofructokinase, liver	1.06	0.8032
	PGAM2	NM_000290	Phosphoglycerate mutase 2 (muscle)	**0.28**	0.0007
	PGK1	NM_000291	Phosphoglycerate kinase 1	0.76	0.3228
	PGK2	NM_138733	Phosphoglycerate kinase 2	0.85	0.6522
	PGM1	NM_002633	Phosphoglucomutase 1	2.04	0.1234
	PGM2	NM_018290	Phosphoglucomutase 2	0.95	0.7028
	PGM3	NM_015599	Phosphoglucomutase 3	0.93	0.7493
	PKLR	NM_000298	Pyruvate kinase, liver and RBC	7.07	0.0604
	PKM2	NM_002654	Pyruvate kinase, muscle 2	0.78	0.4184
	TPI1	NM_000365	Triosephosphate isomerase 1	0.75	0.4871
	SLC2A1	NM_006516	Solute carrier family 2 (facilitated glucose transporter), member 1	1.14	0.1090
	SLC2A2	NM_000340	Solute carrier family 2 (facilitated glucose transporter), member 2	0.67	0.0541
	SLC2A3	NM_006931	Solute carrier family 2 (facilitated glucose transporter), member 3	0.77	0.2152
	SLC2A4	NM_001042	Solute carrier family 2 (facilitated glucose transporter), member 4	2.18	0.1921
	PDK1	NM_002610	Pyruvate dehydrogenase kinase, isozyme 1	0.68	0.2717
	PDK2	NM_002611	Pyruvate dehydrogenase kinase, isozyme 2	1.64	0.1585

**Pyruvate dehydrogenase regulation**	***PDK3***	NM_005391	Pyruvate dehydrogenase kinase, isozyme 3	1.02	0.9694
	PDK4	NM_002612	Pyruvate dehydrogenase kinase, isozyme 4	**3.57**	0.0436
	PDP2	NM_020786	Pyruvate dehyrogenase phosphatase catalytic subunit 2	1.34	0.5304
	PDPR	NM_017990	Pyruvate dehydrogenase phosphatase regulatory subunit	1.09	0.6094
**Pyruvate dehydrogenase complex**	***DLAT***	NM_001931	Dihydrolipoamide S-acetyltransferase	0.94	0.8611
	DLD	NM_000108	Dihydrolipoamide dehydrogenase	1.52	0.1825
	DLST	NM_001933	Dihydrolipoamide S-succinyltransferase	1.43	0.1666
	PDHA1	NM_000284	Pyruvate dehydrogenase (lipoamide) alpha 1	1.21	0.1399
	PDHB	NM_000925	Pyruvate dehydrogenase (lipoamide) beta	**1.33**	0.0121
**TCA cycle**	***ACLY***	NM_001096	ATP citrate lyase	**1.36**	0.0456
	ACO1	NM_002197	Aconitase 1, soluble	**1.61**	0.0019
	ACO2	NM_001098	Aconitase 2, mitochondrial	2.16	0.2250
	CS	NM_004077	Citrate synthase	1.14	0.6518
	FH	NM_000143	Fumarate hydratase	0.98	0.8067
	IDH1	NM_005896	Isocitrate dehydrogenase 1 (NADP+), soluble	1.28	0.1670
	IDH2	NM_002168	Isocitrate dehydrogenase 2 (NADP+), mitochondrial	1.00	0.9803
	IDH3A	NM_005530	Isocitrate dehydrogenase 3 (NAD+) alpha	1.44	0.1534
	IDH3B	NM_174856	Isocitrate dehydrogenase 3 (NAD+) beta	1.36	0.1599
	IDH3G	NM_174869	Isocitrate dehydrogenase 3 (NAD+) gamma	1.16	0.2548
	MDH1	NM_005917	Malate dehydrogenase 1, NAD (soluble)	1.05	0.4306
	MDH1B	NM_001039845	Malate dehydrogenase 1B, NAD (soluble)	1.66	0.3292
	MDH2	NM_005918	Malate dehydrogenase 2, NAD (mitochondrial)	1.36	0.3681
	OGDH	NM_002541	Oxoglutarate dehydrogenase	1.54	0.1297
	PC	NM_000920	Pyruvate carboxylase	1.17	0.2044
	PCK1	NM_002591	Phosphoenolpyruvate carboxykinase 1 (soluble)	1.47	0.4258
	PCK2	NM_004563	Phosphoenolpyruvate carboxykinase 2	0.99	0.9723
	SDHA	NM_004168	Succinate dehydrogenase complex, subunit A, flavoprotein (Fp)	1.21	0.4673
	SDHB	NM_003000	Succinate dehydrogenase complex, subunit B, iron sulfur (Ip)	1.17	0.2019
	SDHC	NM_003001	Succinate dehydrogenase complex, subunit C, integral membrane protein	1.92	0.0836
	SDHD	NM_003001	Succinate dehydrogenase complex, subunit D, integral membrane protein	1.21	0.4810
	SUCLA2	NM_003850	Succinate-CoA ligase, ADP-forming, beta subunit	1.22	0.3138
	SUCLG1	NM_003849	Succinate-CoA ligase, alpha subunit	0.84	0.5854
	SUCLG2	NM_003848	Succinate-CoA ligase, GDP-forming, beta subunit	1.23	0.5511
**Glutamine Metabolism**	***GLS1***	NM_014905	Glutaminase 1	1.21	0.0995
	GLS2	NM_013267	Glutaminase 2	N/A	N/A
	SLC1A5	NM_005628	Solute carrier family 1 (neutral amino acid transporter), member 5	0.97	0.9588
	SLC7A5	NM_003486	Solute carrier family 7 (amino acid transporter light chain, L system), member 5	**0.64**	0.0680

List of analyzed genes. Expression in fold comparing TβT-Hep3B to Hep3B. $p<0.05$ was considered significant. Values <1 indicate lower expression (green shading when significant) and values >1 indicate higher expression (red shading when significant) as compared to Hep3B. N/A indicates Ct values >35 and was considered as non-detectable expression.
